# Insights into Api m 10‐Isoforms and Splice Variants: More Than One Major IgE‐Binding Epitope

**DOI:** 10.1002/clt2.70151

**Published:** 2026-03-06

**Authors:** Kathrin Elisabeth Paulus‐Tremel, Michelle Beatrice Wolff, Natalija Novak, Nicola Wagner, Alisa Landgraf, Stefan Schülke, Thomas Holzhauser, Vera Mahler

**Affiliations:** ^1^ Division Allergology Paul‐Ehrlich‐Institut Langen Germany; ^2^ Faculty of Life Sciences: Food, Nutrition and Health University Bayreuth Kulmbach Germany; ^3^ Centre for Skin Diseases, Department of Dermatology and Allergy University Hospital Bonn Bonn Germany; ^4^ Department of Dermatology University Hospital Erlangen Erlangen Germany; ^5^ Friedrich‐Alexander‐Universität Erlangen‐Nürnberg (FAU) Erlangen Germany

**Keywords:** epitope mapping, honey bee venom allergy, peptide array, secondary structure, venom immunotherapy (VIT)

## Abstract

**Background:**

Honey bee venom (HBV) often triggers severe IgE‐mediated allergies. The major allergen icarapin (Api m 10) has attracted attention due to low occurrence in some HBV immunotherapy products. Despite being a major allergen, little is known about the Api m 10 structure and IgE‐binding regions. This study aimed to characterize its IgE‐binding epitopes and structure in more detail.

**Methods:**

Overlapping Api m 10‐specific peptides covering the sequences of the 11 known Api m 10‐isoforms and variants were synthesized and spotted on microarray slides (15 amino acids (AA), off‐set: 4 AA). Sera from 28 HBV‐allergic patients with detectable Api m 10‐specific IgE were used to characterize the distinct IgE‐binding profiles to all Api m 10‐variants. Sera from ten Api m 10‐immunized BALB/c mice were used to investigate possible shared epitopes between humans and mice. All Api m 10‐variants were investigated for secondary structural elements via circular dichroism spectroscopy and potential aggregation via dynamic light scattering.

**Results:**

We identified 7 different linear IgE‐binding motifs. All 28 patients' sera displayed IgE‐binding to one specific area (present in Api m 10‐isoforms 1 and 2 and putative splice variants 3, 4, 6), indicating a major IgE‐epitope. IgE‐inhibition provided evidence that the major epitope makes up less than 50% of the total IgE‐binding capacity, suggesting that additional (most likely conformational) IgE‐epitopes play an important role in Api m 10‐sensitization. Api m 10‐specific murine IgG and human IgE both predominantly bind to seven different AA‐motifs, of which six are identical between both species. Api m 10‐isoforms 1 and 2 displayed secondary structural elements and appeared to be aggregated.

**Conclusion:**

The structural, clinical and preclinical insights into Api m 10 and its immunodominant epitopes gained in this study provide substantial insights for the future development of active and passive VIT as well as further treatment approaches.

## Graphical abstract

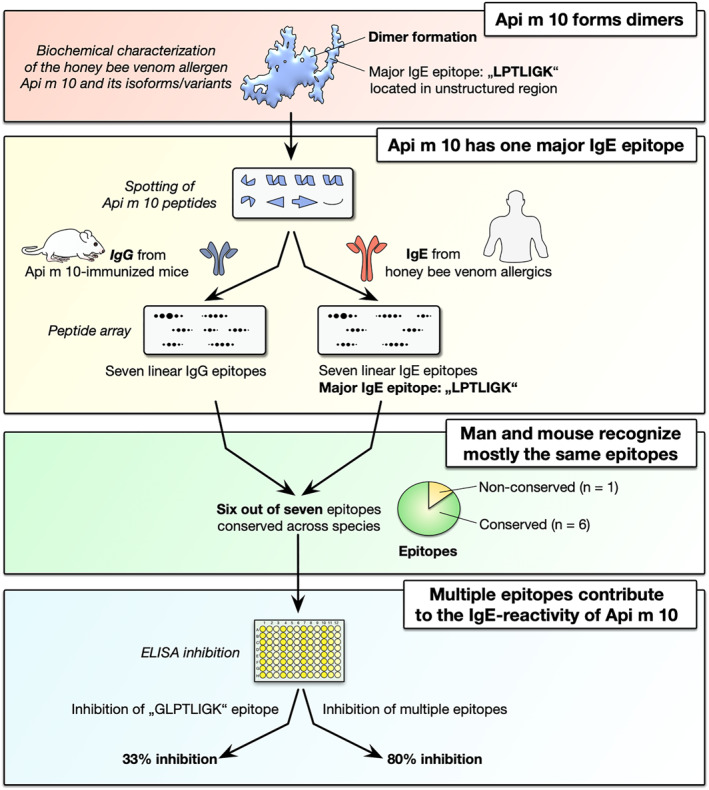

**Abbreviations**
AA: amino acids
AIT: allergen immunotherapy
CBD: chitin‐binding domain
CD: circular dichroism
DLS: dynamic light scattering
DMSO: dimethyl sulfoxide
EB: IgE‐binding site
ECL: enhanced chemiluminescence
GS‐Linker: glycine‐serine linker
HBV: Honey bee venom
HRP: horseradish peroxidase
R_H_
: hydrodynamic radius
SHS: short homologous sequences
VIT: venom immunotherapy
YJV: yellow jacket venom

## 1 Introduction

Insect venoms (as supplied by bee and wasp stings) belong to the most common triggers of serious allergic reactions [1]. Besides toxic reactions, Hymenoptera stings of honey bee (*Apis mellifera*) and the common wasp (*Vespula vulgaris*) may cause local allergic or systemic anaphylactic reactions. Large local reactions are defined as swelling around the puncture site exceeding 10 cm in diameter and lasting longer than 24 h [2]. The prevalence of local reactions to Hymenoptera venom ranges from 2.4% to 26.4% in the general population and up to 38% in beekeepers [3]. According to population‐based epidemiological studies, systemic allergic reactions after insect stings occur in 0.3%–8.9% of the population [4]. The share of systemic Grade IV reactions (anaphylaxis) according to the Mueller Classification [5], within the group of patients with systemic reactions varied from 0.3% to 42.8% in four epidemiological studies [4].

Honey bee venom (HBV) is a complex mixture of both, toxic and allergenic components which, among other things, facilitate a rapid spread of the toxin in the tissue. One honey bee injects up to 140 μg venom per sting with a protein content of about 59 μg protein [6]. To date, 12 HBV allergens have been characterized and included in the official allergen database of the WHO/IUIS subcommittee on allergen nomenclature (https://allergen.org/; last accessed 21st April 2025). Major allergens of HBV are phospholipase A2 (Api m 1), hyaluronidase (Api m 2), acid phosphatase (Api m 3), dipeptidylpeptidase IV (Api m 5) and icarapin (Api m 10) [7]. Icarapin was first discovered in 2005 through proteome studies of bee venom as a carbohydrate‐rich protein of which the function could not be assigned [8, 9]. Shortly afterward, icarapin was further characterized and its role as an allergen has been demonstrated by the detection of IgE‐reactivity of bee venom allergic sera [10]. Api m 10 possesses a 19 amino acids (AA) long signal peptide on its N‐terminus, which is typical for secreted proteins. The natural protein is 223 AA long (including the signal peptide) and has a molecular weight of 55 kDa of which 35 kDa result from the protein itself, and the remaining molecular weight is attributed to posttranslational glycosylation [11].

In the last years, Api m 10 received special attention, because of its suspected absence or underrepresentation in some HBV therapy allergen products [11, 12, 13, 14]. In some of these studies, a putative underrepresentation of this major allergen was found using immunoblotting as detection method, which was explained by the instability of the glycoprotein [10, 11, 13, 14]. Consequently, Api m 10 underrepresentation in therapy allergens and a predominant Api m 10‐sensitization in HBV allergic patients were interpreted as potential predictors of treatment failure in the scientific literature [12, 13, 14], although supportive evidence from prospective clinical studies is lacking. Assumingly, processing of venom immunotherapy (VIT) products during the manufacturing process leads to the loss or reduction of particularly small components of HBV [10] and therefore may influence the overall composition. This is supported by the recent quantification of Api m 10 content across authorized products in Germany, which showed Api m 10 concentrations in authorized HBV VIT‐products from 0.03 to 0.37 ng Api m 10/μg total protein, whereas the Api m 10 content in crude HBV was 1.58–1.92 ng/μg total protein [15].

Component‐resolved analysis of HBV allergens revealed that in addition to Api m 10 other major HBV allergens, such as Api m 3 and Api m 5, were underrepresented in certain therapy allergen products [14]. Allergen concentrations varied not only between different suppliers but in some products also from batch to batch [14]. Frick and coworkers demonstrated in a retrospective study including 115 VIT‐treated HBV allergic patients that patients with a predominant sensitization to Api m 10 had a higher risk of an unsuccessful VIT [13]. In the same study, the presence of the major allergens Api m 1, Api m 3, and Api m 10 in five HBV VIT‐products was investigated. Api m 1 and Api m 3 could be detected in all preparations. In contrast, Api m 10 was underrepresented or absent in 3 out of 5 HBV VIT‐products. Investigations on specific Api m 10‐specific IgG_4_ responses in 109 patients who had received VIT demonstrated significant induction of sIgG_4_ only in those patients (*n* = 50) treated with HBV VIT‐products that contained detectable amounts of Api m 10 [13].

Although Api m 10 has been discovered more than 15 years ago and is considered a major allergen, its function still remains unknown, possibly due to its carbohydrate‐rich and inherently instable nature [9, 10]. In line with this, little is known about its structure and its IgE‐binding domains. Recently, a major IgE‐epitope of Api m 10 was identified in one of its isoforms [16]. Two Api m 10 transcripts generated by alternative splicing were described, resulting in two isoforms: variant 1 (Acc. No. Q5EF78‐1) and variant 2 (Acc. No. Q5EF78‐2) [11, 12]. Both show IgE reactivity independent of cross‐reactive carbohydrate determinants. The difference between the two isoforms is that Api m 10 isoform 2 lacks a four amino acid region (Δ_71–74_, AA sequence: SAIS) compared to Api m 10 isoform 1 [10, 11]. This difference arises from the use of an alternative splice acceptor site in exon 3, following the canonical GT‐AG splicing rule at the end of exon 2 [17]. As a result, the two isoforms differ by only 12 base pairs, leading to a change in the amino acid sequence from “SAISA” (isoform 1) to “T” in isoform 2. In 2015, Van Vaerenbergh and coworkers demonstrated via RT‐PCR that in addition to the already known two Api m 10‐isoforms at least nine additional mRNA transcripts for Api m 10‐variants are produced in venom glands [18]. The gene coding for Api m 10 is located on chromosome LG1 and consists of 4 exons. Two different mechanisms were identified that seem to be responsible for the generation of the different variants: mRNA transcripts of Api m 10‐variants 1 and 2 are generated by an alternative splicing site in exon 3 (as described above) [18]. In contrast, the remaining discovered variants are chimeric transcripts, which differ from conventionally spliced mRNA isoforms because they are produced by connecting exons from two or more different gene transcripts. Instead of splicing sites, short homologous sequences (SHS) are present at the junctions of the original sequence. The Api m 10 gene contains several different SHS in exon 2 that are linked to SHS of exon 3 or exon 4 and have a length of 5–9 base pairs. In the transcripts of variants 8 to 11, this mechanism results in a shift of the reading frame, producing premature stop codons that result in C‐terminally shortened proteins. Deprived from the signal peptide, with 204 AA, Api m 10 isoform 1 forms the longest isoform, closely followed by Api m 10 isoform 2 with 200 AA. The estimated lengths of Api m 10 splice variants 8 to 11 are between 41 and 12 AA. Whether all these variants are translated into proteins in the venom glands has still to be confirmed [18]. Bottom‐up proteomics which analyses peptides generated by proteolytic digestion was proposed for the unequivocal detection of Api m 10‐isoforms in HBV for the analysis of isoform‐specific transition peptides [18, 19]. However, besides the initial description of the different Api m 10 splice variants [18], no additional data have been reported on their transcript levels and potential in vivo translation. Especially, the relevance of the strongly truncated Api m 10 splice variants 8 to 11, which were already initially suggested to potentially be loss‐of‐function mutants [18], remains an open question. Consequently, in order to fully define the IgE‐binding profile of Api m 10, our analyses included all known splice variants but focused on the Api m 10‐isoforms 1 and 2.

Aim of this study was to examine the structure of Api m 10 and IgE‐binding epitopes of previously described Api m 10‐variants in detail using linear epitope mapping in multiple‐peptide microarrays.

## 2 Material and Methods

### 2.1 Patients and Sera

Patients were included based on their case history indicative for insect venom allergy (classified according to Mueller [5]) and a positive response to HBV in diagnostic skin tests and/or specific IgE against HBV (and/or its components) (Table 1). Api m 10 sensitivity of these patients was confirmed by Api m 10‐specific serum IgE (≥ 0.1 kU_A_/L) using the ImmuoCAP‐FEIA (Thermo Fisher, Uppsala, Sweden). Sera from HBV allergic patients showing no sensitization (< 0.1 kU_A_/L) to Api m 10 (*n* = 2; L7 and L9 in Table 1) and from non‐allergic persons served as negative controls (*n* = 3; N1‐3 in Table 1). Four HBV‐sensitized patients included in our cohort displayed only local reactions (Grade 0 according to Mueller). All patients were treated in Germany, approval of the local ethics committee for this study and written informed consent of all participants were obtained. Although geographical differences may exist concerning sensitization profiles, sensitization rates for Api m 10 and patient selection for VIT, these are likely to play only a minor role for the characterization of IgE recognition sites of Api m 10. This is in line with Monograph 2623 (Hymenoptera venoms for allergen products) [21]: To determine the total allergenic activity of a VIT‐product, testing the inhibition of its binding capacity of specific immunoglobulin *E* antibodies is required (without any regional segregation).

**TABLE 1:** Clinical and serological characterization of patients and controls.

Demographics			Clinical characterization			ImmunoCAP (kU_A_/L)				VIT
ID	Age/Sex	Profession	Grading of allergic symptoms (according to Mueller)	Eliciting venom based on clinical history: X		HBV	Api m 1	Api m 10	Total IgE	
				OR: sensitization only: #						
				HBV	YJV					
L1	53/m	Biochemist	Grade 0	X	—	1.62	0.19	1.25	1026	No
L2	43/f	Industrial mechanic	Grade II	X	X	3.86	0.39	0.43	412	No
L3	83/f	Pensioner	Grade II	X	X	0.73	< 0.10	0.58	127	No
L4	75/m	Merchant	Grade 0	X	—	0.74	0.47	0.37	236	No
L5	38/f	Marketing	Grade 0	X	X	13.6	1.37	0.35	1090	No
L6	65/m	Machine operator	Grade I	X	—	8.81	0.19	0.73	130	HBV
L7	65/f	Housewife	Grade II	#	X	2.59	< 0.10	0.00	71.8	No
L8	62/f	Teacher	Anaphylaxis Grade 2 according to Ring and Messmer without identified elicitor. Type I‐sensitization to HBV and YJV w/o confirmed connection to the anaphylactic reaction. No conclusive history for insect sting	#	#	6.63	0.80	1.12	245	No
L9	62/m	Wood processor	Grade III	#	X	0.78	< 0.10	0.03	76.2	YJV
L10	33/m	Cleaner	Grade III	X	X	26.6	26.0	1.23	196	HBV
L11	23/f	Student	Grade II	#	X	1.67	0.26	0.34	392	YJV
E1	49/f	Biologist	Grade III	X	—	10.5	3.02	12.4	33.4	HBV
E2	15/m	Student, beekeeper	Grade II	X	—	21.9	8.31	3.68	770	HBV
E3	54/f	Teacher, beekeeper	Grade I	X	—	> 100	22.2	4.85	842	No
E4	16/f	Student	Grade II	X	—	37.9	6.34	31.00	1761	HBV
E5	66/m	Pensioner	Grade I	X	X	6.49	0.7	5.14	327	HBV; YJV
E6	31/f	Teacher	Grade II	X	—	2.52	1.47	3.06	31.3	HBV
E7	47/m	Engineer, beekeeper	Grade III	X	—	11.9	5.58	3.24	329	HBV
E8	45/m	Carpenter	Grade IV	X	X	29	13.9	7.69	629	HBV; YJV
E9	23/m	Industrial sales representative	Grade II	X	—	21.7	4.14	4.35	1976	HBV
E10	23/f	Student	Grade 0	X	—	38.4	21.1	8.30	872	No
B1	58/m	Beekeeper	Grade II	X	—	> 100	55.4	1.04	221.0	HBV
B2	58/m	n.k.	Grade II	#	X	< 0.10	0.02	0.76	758.0	YJV
B3	27/f	n.k.	Grade III	X	—	9.77	2.26	7.18	39.6	HBV
B4	50/m	n.k.	Grade II	X	—	3.46	1.38	0.5	270.0	HBV
B5	25/m	Municipal worker	Grade II	X	X	68.2	11.7	30.1	692.0	YJV
B6	50/m	Beekeeper	Grade III	X	X	69.2	15.7	0.52	171.0	HBV
B7	59/m	n.k.	Grade III	X	X	2.09	0.1	0.81	185.0	YJV
B8	41/f	n.k.	Grade III	X	X	0.952	0.12	0.54	27.3	HBV
B9	32/f	n.k.	Grade II	X	X	1.12	0.45	1.35	285.0	YJV
N1	28/f	Biomolecular engineer	None	—	—	< 0.10	< 0.10	< 0.10	32.2	No
N2	56/f	Medical doctor	None	—	—	< 0.10	< 0.10	< 0.10	9.7	No
N3	40/f	Biologist	None	—	—	< 0.10	< 0.10	< 0.10	< 2.0	No

*Note:* Assay units and reporting scale for ImmunoCAP [20]: Class 0: < 0.1 kU_A_/L, Class 0/1: 0.1–0.34 kU_A_/L, Class 1: 0.35–0.70 kU_A_/L, Class 2: > 0.70–3.5 kU_A_/L, Class 3: > 3.5–17.5 kU_A_/L, Class 4: > 17.5–50 kU_A_/L, Class 5: > 50–100 kU_A_/L, Class 6: > 100 kU_A_/L.

Abbreviations: #, a sting of the respective insect eliciting the allergic symptoms was not recalled; HBV, honey bee venom; n.d., not done; n.k., not known; X, in the clinical history, a sting of the respective insect was recalled as elicitor of allergic symptoms; YJW, yellow jacket venom.

### 2.2 Expression of Api m 10 and Its Splice Variants in *E. coli* for Molecular and Clinical Analysis

For recombinant expression of Api m 10‐isoforms and splice variants, the coding DNA‐sequences of Api m 10‐isoforms 1 and 2 and splice variants 3 to 5 were purchased as synthetic genes from GENEART Gene Synthesis (Thermo Fisher Scientific, Regensburg, Germany). Based on published evidence [9, 15] the decision was made to express mature Api m 10 (without the N‐terminal secretory signal sequence “MKTLGVLFIAAWFIACTHS”). Api m 10‐isoforms 1 and 2 as well as splice variants 3 to 5 were produced as recombinant proteins in *E. coli*. Api m 10 splice variants 6 to 11 as well as “peptide L” (see below) were purchased from Biomatik Corporation (Ontario, Canada) as synthetic peptides. The peptide L contains two copies of the AA‐sequence of an identified immunodominant motif (within the IgE‐binding region EB6), separated by a linker of glycines and serines (GS‐linker), resulting in the sequence: GLPTLIGKGSGGGGSGLPTLIGKG‐NH_2_. The duplicate presentation of the immunodominant motif in one synthetic peptide was based on the experimental observation that Api m 10 dimerizes under native conditions (Supporting Information S1: Figure 3). The GS‐linker was used to spatially separate both identical epitopes and allow for additional flexibility of epitope presentation.

According to the allergen nomenclature for recombinant and synthetic peptides, recombinant allergens are marked by the addition of the prefix “r” (rApi m 10) and synthetic peptides by the addition of the prefix “s” (sApi m 10) [22]. Recombinant proteins were expressed and purified as intein‐fusion protein using a chitin‐binding domain. After chromatographic removal of the self‐cleaving intein stretch, rApi m 10 isoforms/variants were obtained tag‐less according to manufacturer's instructions (NEB‐Biolabs, Frankfurt, Germany).

### 2.3 Membrane Bound Peptide Synthesis and Production of CelluSpot Slides

Synthetic overlapping peptides (15 mer, 4 AA offset), spanning the entire primary sequence of Api m 10, were obtained according to the Merrifield‐synthesis principle [23]. The solid phase for the synthesis was Fmoc‐β‐Alanine derivatized cellulose membrane (384 frame filled derivatized cellulose discs, Intavis AG, Cologne, Germany). After removal of protection groups, the cellulose, carrying peptides, was ether purified, dissolved in 300 μL DMSO and stored at −20°C [25, 26]. The sequences of Api m 10‐specific peptides for peptide microarray are listed in Table 2.

**TABLE 2:** Sequences of Api m 10‐specific peptides for peptide microarray.

Peptide	ID	Isoform 1	ID	Isoform 2	ID	Splice variant 3	ID	Splice variant 4
1	**P1**	**MKTLGVLFIAAWFIA**	P1	MKTLGVLFIAAWFIA	P1	MKTLGVLFIAAWFIA	P1	MKTLGVLFIAAWFIA
2	**P2**	**GVLFIAAWFIACTHS**	P2	GVLFIAAWFIACTHS	P2	GVLFIAAWFIACTHS	P2	GVLFIAAWFIACTHS
3	**P3**	**IAAWFIACTHSFPGA**	P3	IAAWFIACTHSFPGA	P3	IAAWFIACTHSFPGA	P3	IAAWFIACTHSFPGA
4	**P4**	**FIACTHSFPGAHDED**	P4	FIACTHSFPGAHDED	P4	FIACTHSFPGAHDED	P4	FIACTHSFPGAHDED
5	**P5**	**THSFPGAHDEDSKEE**	P5	THSFPGAHDEDSKEE	P5	THSFPGAHDEDSKEE	P5	THSFPGAHDEDSKEE
6	**P6**	**PGAHDEDSKEERKNV**	P6	PGAHDEDSKEERKNV	P6	PGAHDEDSKEERKNV	P6	PGAHDEDSKEERKNV
7	**P7**	**DEDSKEERKNVDTVL**	P7	DEDSKEERKNVDTVL	P7	DEDSKEERKNVDTVL	P7	DEDSKEERKNVDTVL
8	**P8**	**KEERKNVDTVLVLPS**	P8	KEERKNVDTVLVLPS	P8	KEERKNVDTVLVLPS	P8	KEERKNVDTVLVLPS
9	**P9**	**KNVDTVLVLPSIERD**	P9	KNVDTVLVLPSIERD	P9	KNVDTVLVLPSIERD	P9	KNVDTVLVLPSIERD
10	**P10**	**TVLVLPSIERDQMMA**	P10	TVLVLPSIERDQMMA	P10	TVLVLPSIERDQMMA	P10	TVLVLPSIERDQMMA
11	**P11**	**LPSIERDQMMAATFD**	P11	LPSIERDQMMAATFD	**P58**	**LPSIERDQMMAGILS**	P11	LPSIERDQMMAATFD
12	**P12**	**ERDQMMAATFDFPSL**	P12	ERDQMMAATFDFPSL	**P59**	**ERDQMMAGILSRIPE**	P12	ERDQMMAATFDFPSL
13	**P13**	**MMAATFDFPSLSFED**	P13	MMAATFDFPSLSFED	**P60**	**MMAGILSRIPEQGVV**	P13	MMAATFDFPSLSFED
14	**P14**	**TFDFPSLSFEDSDEG**	P14	TFDFPSLSFEDSDEG	**P61**	**ILSRIPEQGVVNWNK**	**P88**	**TFDFPSLSFEDSDVT**
15	**P15**	**PSLSFEDSDEGSNWN**	P15	PSLSFEDSDEGSNWN	**P62**	**IPEQGVVNWNKIPEG**	**P89**	**PSLSFEDSDVTTLPT**
16	**P16**	**FEDSDEGSNWNWNTL**	P16	FEDSDEGSNWNWNTL	**P63**	**GVVNWNKIPEGANTT**	**P90**	**FEDSDVTTLPTLIGK**
17	**P17**	**DEGSNWNWNTLLRPN**	P17	DEGSNWNWNTLLRPN	**P64**	**WNKIPEGANTTSTTK**	P80	DVTTLPTLIGKNETS
18	**P18**	**NWNWNTLLRPNFLDG**	P18	NWNWNTLLRPNFLDG	**P65**	**PEGANTTSTTKIIDG**	P81	LPTLIGKNETSTQSS
19	**P19**	**NTLLRPNFLDGWYQT**	P19	NTLLRPNFLDGWYQT	**P66**	**NTTSTTKIIDGHVVT**	P82	IGKNETSTQSSRSVE
20	**P20**	**RPNFLDGWYQTLQSA**	**P54**	**RPNFLDGWYQTLQTH**	**P67**	**TTKIIDGHVVTINET**	P83	ETSTQSSRSVESVED
21	**P21**	**LDGWYQTLQSAISAH**	**P55**	**LDGWYQTLQTHMKKV**	**P68**	**IDGHVVTINETTYTD**	P84	QSSRSVESVEDFDNE
22	**P22**	**YQTLQSAISAHMKKV**	**P56**	**YQTLQTHMKKVREQM**	**P69**	**VVTINETTYTDGSDD**	P85	SVESVEDFDNEIPKN
23	**P23**	**QSAISAHMKKVREQM**	**P57**	**QTHMKKVREQMAGIL**	**P70**	**NETTYTDGSDDYSTL**	P86	VEDFDNEIPKNQGDV
24	**P24**	**SAHMKKVREQMAGIL**	P25	KKVREQMAGILSRIP	**P71**	**YTDGSDDYSTLIRVR**	P87	FDNEIPKNQGDVLTA
25	**P25**	**KKVREQMAGILSRIP**	P26	EQMAGILSRIPEQGV	**P72**	**SDDYSTLIRVRVIDV**		
26	**P26**	**EQMAGILSRIPEQGV**	P27	GILSRIPEQGVVNWN	**P73**	**STLIRVRVIDVRPQN**		
27	**P27**	**GILSRIPEQGVVNWN**	P28	RIPEQGVVNWNKIPE	**P74**	**RVRVIDVRPQNETIL**		
28	**P28**	**RIPEQGVVNWNKIPE**	P29	QGVVNWNKIPEGANT	**P75**	**IDVRPQNETILTTVS**		
29	**P29**	**QGVVNWNKIPEGANT**	P30	NWNKIPEGANTTSTT	**P76**	**PQNETILTTVSSEAD**		
30	**P30**	**NWNKIPEGANTTSTT**	P31	IPEGANTTSTTKIID	**P77**	**TILTTVSSEADSDVT**		
31	**P31**	**IPEGANTTSTTKIID**	P32	ANTTSTTKIIDGHVV	**P78**	**TVSSEADSDVTTLPT**		
32	**P32**	**ANTTSTTKIIDGHVV**	P33	STTKIIDGHVVTINE	**P79**	**EADSDVTTLPTLIGK**		
33	**P33**	**STTKIIDGHVVTINE**	P34	IIDGHVVTINETTYT	**P80**	**DVTTLPTLIGKNETS**		
34	**P34**	**IIDGHVVTINETTYT**	P35	HVVTINETTYTDGSD	**P81**	**LPTLIGKNETSTQSS**		
35	**P35**	**HVVTINETTYTDGSD**	P36	INETTYTDGSDDYST	**P82**	**IGKNETSTQSSRSVE**		
36	**P36**	**INETTYTDGSDDYST**	P37	TYTDGSDDYSTLIRV	**P83**	**ETSTQSSRSVESVED**		
37	**P37**	**TYTDGSDDYSTLIRV**	P38	GSDDYSTLIRVRVID	**P84**	**QSSRSVESVEDFDNE**		
38	**P38**	**GSDDYSTLIRVRVID**	P39	YSTLIRVRVIDVRPQ	**P85**	**SVESVEDFDNEIPKN**		
39	**P39**	**YSTLIRVRVIDVRPQ**	P40	IRVRVIDVRPQNETI	**P86**	**VEDFDNEIPKNQGDV**		
40	**P40**	**IRVRVIDVRPQNETI**	P41	VIDVRPQNETILTTV	**P87**	**FDNEIPKNQGDVLTA**		
41	**P41**	**VIDVRPQNETILTTV**	P42	RPQNETILTTVSSEA				
42	**P42**	**RPQNETILTTVSSEA**	P43	ETILTTVSSEADSDV				
43	**P43**	**ETILTTVSSEADSDV**	P44	TTVSSEADSDVTTLP				
44	**P44**	**TTVSSEADSDVTTLP**	P45	SEADSDVTTLPTLIG				
45	**P45**	**SEADSDVTTLPTLIG**	P46	SDVTTLPTLIGKNET				
46	**P46**	**SDVTTLPTLIGKNET**	P47	TLPTLIGKNETSTQS				
47	**P47**	**TLPTLIGKNETSTQS**	P48	LIGKNETSTQSSRSV				
48	**P48**	**LIGKNETSTQSSRSV**	P49	NETSTQSSRSVESVE				
49	**P49**	**NETSTQSSRSVESVE**	P50	TQSSRSVESVEDFDN				
50	**P50**	**TQSSRSVESVEDFDN**	P51	RSVESVEDFDNEIPK				
51	**P51**	**RSVESVEDFDNEIPK**	P52	SVEDFDNEIPKNQGD				
52	**P52**	**SVEDFDNEIPKNQGD**	P53	FDNEIPKNQGDVLTA				
53	**P53**	**FDNEIPKNQGDVLTA**						

Peptide	ID	Splice variant 5	ID	Splice variant 6	ID	Splice variant 7	ID	Splice variant 8
1	P1	MKTLGVLFIAAWFIA	P1	MKTLGVLFIAAWFIA	P1	MKTLGVLFIAAWFIA	P1	MKTLGVLFIAAWFIA
2	P2	GVLFIAAWFIACTHS	P2	GVLFIAAWFIACTHS	P2	GVLFIAAWFIACTHS	P2	GVLFIAAWFIACTHS
3	P3	IAAWFIACTHSFPGA	P3	IAAWFIACTHSFPGA	P3	IAAWFIACTHSFPGA	P3	IAAWFIACTHSFPGA
4	P4	FIACTHSFPGAHDED	**P94**	**FIACTHSFPGAHDVT**	P4	FIACTHSFPGAHDED	P4	FIACTHSFPGAHDED
5	P5	THSFPGAHDEDSKEE	**P95**	**THSFPGAHDVTTLPT**	P5	THSFPGAHDEDSKEE	P5	THSFPGAHDEDSKEE
6	P6	PGAHDEDSKEERKNV	**P96**	**PGAHDVTTLPTLIGK**	**P97**	**PGAHDEDSKEERKNE**	**P106**	**PGAHDEDSKEERMRP**
7	P7	DEDSKEERKNVDTVL	P80	DVTTLPTLIGKNETS	**P98**	**DEDSKEERKNETSTQ**	**P107**	**DEDSKEERMRPAPNL**
8	P8	KEERKNVDTVLVLPS	P81	LPTLIGKNETSTQSS	**P99**	**KEERKNETSTQSSRS**	**P108**	**KEERMRPAPNLQGVW**
9	P9	KNVDTVLVLPSIERD	P82	IGKNETSTQSSRSVE	**P100**	**KNETSTQSSRSVESV**	**P109**	**MRPAPNLQGVWKASR**
10	P10	TVLVLPSIERDQMMA	P83	ETSTQSSRSVESVED	**P101**	**STQSSRSVESVEDFD**	**P110**	**PNLQGVWKASRISTT**
11	P11	LPSIERDQMMAATFD	P84	QSSRSVESVEDFDNE	**P102**	**SRSVESVEDFDNEIP**	**P111**	**GVWKASRISTTRYRR**
12	P12	ERDQMMAATFDFPSL	P85	SVESVEDFDNEIPKN	**P103**	**ESVEDFDNEIPKNQG**	**P112**	**ASRISTTRYRRTKEM**
13	P13	MMAATFDFPSLSFED	P86	VEDFDNEIPKNQGDV	**P104**	**DFDNEIPKNQGDVLT**	**P113**	**SRISTTRYRRTKEMY**
14	**P91**	**TFDFPSLSFEDFDNE**	P87	FDNEIPKNQGDVLTA	**P105**	**FDNEIPKNQGDVLTA**		
15	**P92**	**PSLSFEDFDNEIPKN**						
16	**P93**	**FEDFDNEIPKNQGDV**						
17	P87	FDNEIPKNQGDVLTA						

Peptide	ID	Splice variant 9	ID	Splice variant 10	ID	Splice variant 11		
1	P1	MKTLGVLFIAAWFIA	P1	MKTLGVLFIAAWFIA	P1	MKTLGVLFIAAWFIA		
2	P2	GVLFIAAWFIACTHS	P2	GVLFIAAWFIACTHS	P2	GVLFIAAWFIACTHS		
3	P3	IAAWFIACTHSFPGA	P3	IAAWFIACTHSFPGA	P3	IAAWFIACTHSFPGA		
4	P4	FIACTHSFPGAHDED	P4	FIACTHSFPGAHDED	P4	FIACTHSFPGAHDED		
5	**P114**	**THSFPGAHDEDSKER**	P5	THSFPGAHDEDSKEE	P120	**THSFPGAHDEDSKVL**		
6	**P115**	**PGAHDEDSKERTLPL**	P6	PGAHDEDSKEERKNV				
7	**P116**	**DEDSKERTLPLPPRS**	**P119**	**HDEDSKEERKNVDTW**				
8	**P117**	**KERTLPLPPRSSMDT**						
9	**P118**	**ERTLPLPPRSSMDTW**						

*Note:* The peptides highlighted in bold were synthesized and used for spotting of the peptide arrays.

Using the CelluSpot technique, multi‐peptide arrays were generated using a slide spotting robot (Intavis AG, Cologne, Germany). The technique and its performance have been previously described elsewhere [24]. In brief, each peptide was spotted in quadruplicate on a coated slide (Lasertab Markers, Brady, Egelsbach, Germany). For control purposes, biotinylated peptides (Biotin‐AANWSHPQFEKAA) were additionally applied to the slide. DMSO‐spotting was applied on empty positions on the slide. The spotted slides were heated for 1 hour at 75°C and stored dry at 4°C [25, 26].

### 2.4 IgE‐Immunodetection and ‐Inhibition on Peptide Microarrays

**FIGURE 1:**
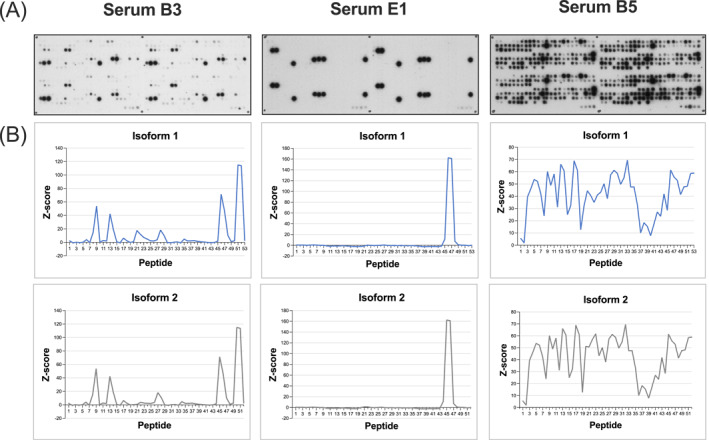
IgE‐reactive peptides of Api m 10 in peptide microarrays. (A) Representative Api m 10‐specific CelluSpot slides of three Api m 10‐reactive patients' sera. Left: Serum B3 Middle: Serum E1, Right: Serum B5. (B) The data of the Api m 10 peptide arrays were normalized by calculating the Z‐score and the values for the individual peptides were plotted according to the sequences of the Api m 10‐isoforms from the respective N‐ to C‐termini. The IgE‐recognition profile for Api m 10 isoform 1 and 2 is presented.

First, CelluSpot Slides were incubated for 2 h in blocking buffer, and subsequently overnight with 45 μL individual patients' sera (undiluted) in a wet incubation chamber. Mouse anti‐human IgE Fc antibody conjugated with horseradish peroxidase (HRP) (Southern Biotechnology Associates Inc., Birmingham, USA) was used as secondary antibody and final immunodetection was done using the ECL system LumiGlo Reserve (SeraCare, Milford, USA) according to the manufacturer's instruction. The exposure times for the X‐ray films varied between 15 and 60 s (the variation of exposure times is owing to the established detection system: Detection on x‐ray films is highly sensitive but only covers a small dynamic range. In order to avoid over‐exposure for highly potent IgE sera or underexposure for sera with low levels of specific IgE, an appropriate window of exposure is found by variation of exposure times within the indicated range. The developed X‐ray films were digitalized with a transmitted light scanner and converted into a 16‐bit‐tiff format with 600 dpi. The scanned peptide array slides were adapted to a size of 1796 × 615 pixels, inverted and the individual spots were quantified using the MAPIX software (Innopsys, Carbonne, France). Z‐scores of chemiluminescence signals of IgE‐immunodetection on arrays were calculated for each peptide and each patient serum to obtain signal normalization for the reason of comparability between patients' IgE‐binding capacity. Z‐scores of peptide/patient‐specific signals were calculated as the multiple of background noise, that is the standard deviation of the stable mean of blanks (DMSO spots). Details for calculation were published previously [25]. We emphasize that Z‐scores obtained by diluted serum samples or varying exposure times should not be considered quantitative but do allow for semi‐quantitative comparison of peptide‐IgE‐binding within and between patients. Moreover, the microarray technique is a screening tool. All findings should be verified in subsequent studies because minor variations in signal intensity may not account for substantial differences in findings. The spotting layout of 120 Api m 10‐specific peptides in quadruplicates is displayed in the (Supporting Information S1: Figure 1).

The immuno‐inhibitions with the synthetic “peptide L” or rApi m 10 were performed as previously described [25]. In brief, the patients' sera were pre‐incubated either with 20 μg of peptide L or 30 μg of rApi m 10 isoform 1 for 3 hours. Since the serum was diluted by the addition of the inhibitor, the same (non‐inhibited) serum was diluted with blocking buffer at the same ratio for better comparability. All incubations were carried out at ambient temperature.

### 2.5 ELISA Inhibition

Ninety‐six‐well Maxisorb plates (Nunc, Wiesbaden, Germany) were coated overnight at 4°C with 0.5 μg of rApi m 10 isoform 1 diluted in PBS. The plates were blocked with PBST (0.05% Tween 20) + 1% bovine serum albumin (BSA) for 2 h. After three washing steps with PBST, each well was incubated with 100 μL diluted human pool serum (consisting of five Api m 10‐positive sera with high Api m 10‐specific IgE‐levels (ImmunoCAP class 3 or 4); diluted 1:10 with PBS). The pooled sera were pre‐incubated with different concentrations of either Api m 10‐variants or peptide L [100 μg/mL; 10 μg/mL; 1 μg/mL; 0.1 μg/mL; 0.01 μg/mL] for 90 min at room temperature. As a negative control, BSA was applied as sham inhibitor. Subsequently, plates were washed and incubated with mouse anti‐human IgE Fc‐HRP coupled for 1.5 h. After three additional washing cycles, visualization was performed with tetramethylbenzidine and hydrogen peroxide for approximately 5–10 min. The color reaction was stopped with 3 M H_2_SO_4,_ and adsorption was measured at 450 nm. Duplicate determinations were carried out with each sample. Inhibition rates were calculated as a percentage of the values of uninhibited sera under consideration of the non‐specific binding control (no serum, no antibody). The mean of three blank values was subtracted as background signal.

### 2.6 Circular Dichroism Spectroscopy

Far UV circular dichroism (CD) spectra of the rApi m 10 and sApi m 10 variants were acquired at 293 K using a Jasco J‐810 spectropolarimeter (Japan Spectroscopic, Gross‐Umstadt, Germany) at a bandwidth of 1 nm and a sensitivity of 100 mdeg in a 1 mm quartz cuvette. Each measurement comprised the average of 10 repeated scans between 255 and 185 nm at a scanning speed of 50 nm/min. rApi m 10 isoform 1, rApi m 10 isoform 2, rApi m 10 splice variant 3, rApi m 10 splice variant 4 and rApi m 10 splice variant 5 were analyzed at a concentration of 3.3 μM in 10 mM potassium phosphate buffer, pH 7.4. Synthetic peptides sApi m 10 splice variants 6–10 and the linker peptide, peptide L, were analyzed at a concentration of 100 μM in 10 mM KPi, pH 7.4, sApi m 10 splice variant 11 was measured at a concentration of 50 μM. The spectrum of buffer alone was subtracted. Results are shown as mean residual ellipticity.

### 2.7 Dynamic Light Scattering

The dynamic light scattering (DLS) was performed as previously described [26] to determine the hydrodynamic radii (*R*_*H*_) of proteins. Briefly, protein samples with concentrations of at least 5 μM were centrifuged at 4°C and 16,000 *g* for 15 min, and supernatants were analyzed in a Zetasizer Nano‐ZS (Malvern, Herrenberg, Germany) at 25°C and 633 nm wavelength. Three individual measurements with 10 runs each were carried out in UV micro cuvettes (Carl Roth, Karlsruhe, Germany) with a layer thickness of 10 mm.

### 2.8 Mice

Six‐to eight‐week‐old BALB/c mice (5 female and 5 male animals) were purchased (Charles River Laboratories, Sulzfeld, Germany) and housed in the animal facility under specific pathogen free conditions. The mouse model was performed in compliance with German animal protection law (granting authority: RP Darmstadt, Germany, approval no. F107/1064).

Tag‐less rApi m 10 [15] was injected without adjuvant in female and male animals. Immunization consisted of an up‐dosing and a maintenance phase (scheduled doses [μg]: 0.1, 0.2, 0.4, 0.8, 1.6, 3.2, 6.4, 4 × 10, 20, 30, 4 × 50). Subcutaneous injections were administered every 14 days (in parallel with blood collection in the same session) until mice reached the pre‐defined cut‐off level of rApi m 10‐specific total IgG (OD_450_ ≥ 1; at 1:10,000 serum dilution in indirect ELISA) [23]. Furthermore, mice received a final boost immunization using the last applied dose 3 days pre‐sacrifice.

The amount of rApi m 10 that mice received varied based on the time points at which they reached the predetermined cut‐off. The injected doses were 0.1, 0.2, 0.4, 0.8, 1.6, 3.2 μg (last immunization for one female mouse), 6.4 μg (last immunization for two female mice), 10 μg (last immunization for two female mice), 10 μg (last immunization for one male mouse), second dose of 10 μg (last immunization for three male mice), and 30 μg (last immunization for one male mouse) per mouse and injection.

For peptide array analysis, a similar protocol as described above (see section IgE‐immunodetection and ‐inhibition on peptide microarrays) was used to determine Api m 10‐specific epitopes of murine IgG. Female and male mouse sera were pooled separately in equal volumes and diluted 1:10,000 in blocking buffer. Of these, 3 mL were added after blocking and first washing steps to CelluSpot Slides and incubated over night at 4°C. Slides were then incubated with 3 mL of HRP‐labelled anti‐Mouse IgG (γ‐chain specific, #A3673, Sigma‐Aldrich, St. Louis, MO, USA) as secondary antibody diluted 1:40,000 in blocking buffer for 2 h. For detection via chemiluminescence system as described above, slides were exposed for 10–40 s.

### 2.9 Statistical Analysis

The hypothesis of a significant higher IgE inhibition for all five concentrations used for ELISA inhibition was tested with a two‐factorial analysis of variance (ANOVA) with factors inhibitor concentration (0.01, 0.1, 1, 10, and 100) and group using Graphpad Prism (V10.6.0 for Mac). Correction for multiple comparisons was done according to Tukey.

## 3 Results

### 3.1 Patients

In total, 30 HBV allergic patients (Mueller classification Grade 0–Grade IV) [5] were included (Table 1). The median age was 48 years (age range from 15 to 83 years), 53% were male, 47% were female. All patients, except for one patient (L8), had a decisive insect sting‐related history of allergic reactions. 16 patients (53%) displayed double sensitization against HBV and yellow jacket venom (YJV). 21 patients (70%) had undergone VIT (*n* = 13 HBV, 2 HBV and YJV, 6 YJV). Among the 30 HBV allergic patients, 28 (93%) displayed specific IgE against Api m 10 (≥ 0.1 kU/L) in the range between 0.34 kU/L and 31.0 kU/L. Sera of these 28 Api m 10‐sensitized patients were used for epitope mapping of Api m 10. Sera from three non‐allergic donors (N1, N2, N3) and two Api m 10‐negative HBV allergic individuals (L7, L9) were used as negative controls in these investigations.

### 3.2 Epitope Mapping of Api m 10 via Peptide Microarray

**FIGURE 2:**
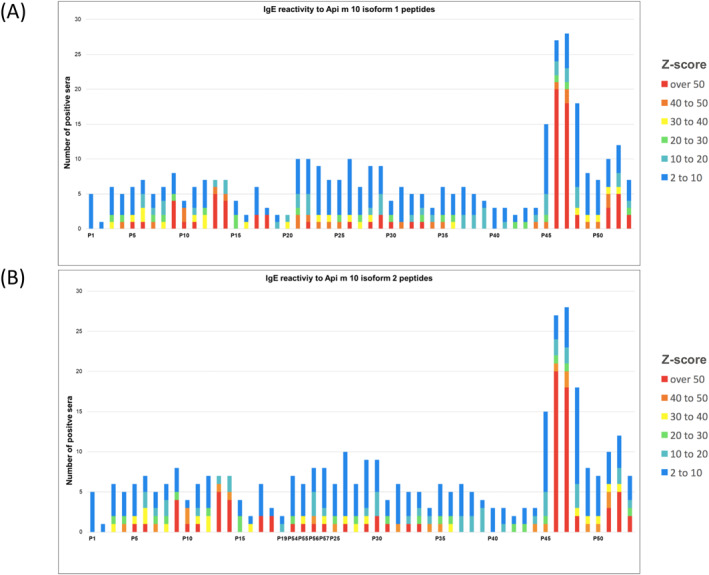
Accumulated Z‐scores of IgE‐binding to Api 10 peptides in 28 Api m 10‐sensitized sera. The accumulated Z‐scores (> 2) of IgE‐binding to Api m10 peptides from 28 patients' sera were plotted against the consecutive sequences of the peptides (15 mers with 4 AA offset) of the two different Api m 10‐isoforms 1 and 2.

Different epitope recognition patterns were observed within the Api m 10‐sensitive human sera. Representative examples of Api m 10‐peptide arrays and the immunodetection of IgE‐epitopes by three Api m 10‐sensitive human sera are shown in Figure 1. The sera B3 (Figure 1A left; 7.18 kU/L Api m 10‐sIgE) and E1 (Figure 1A middle; 12.4 kU/L Api m 10‐sIgE) showed distinct IgE‐reactivity, serum B5 (containing Api m 10‐specific IgE of 30.1 kU/L) detected numerous IgE‐binding peptides (Figure 1A right). Detection signals, normalized as Z‐scores for inter‐array comparability, plotted against the respective Api m 10 peptides are shown in Figure 1B. Serum B3 (Figure 1B left) displayed two IgE recognition sites in each N‐ and C‐terminal region of the Api m 10‐isoforms 1 and 2, which were also present in splice variant 3 (Supporting Information S1: Figure 2A). Api m 10 splice variant 4 consists almost exclusively of these sequence regions and splice variants 6 and 7 also contained a part of the sequence. Peptides 13–15 of splice variant 5 were recognized with a Z‐score of 130 and are localized at the junction that composes the variant 5. Serum E1 (Figure 1 middle; Supporting Information S1: Figure 2B) represents an IgE‐binding profile that was frequently observed among the 28 tested sera. Only one single region at the C‐terminus (peptide 46 and 47 in isoform 1) was detected, which occurs in the Api m 10‐isoforms 1 to 4 and splice variant 6. The serum B5 (Figure 1 right; Supporting Information S1: Figure 2C) showed a strong and complex IgE‐binding pattern incuding nearly all tested peptides. IgE molecules did not or only weakly bind to peptides 1 and 2. These two peptides consisted exclusively of AA of the signal peptide, which is probably cleaved upon secretion of Api m 10 into the venom gland of the bee [10]. No direct correlation between either specific epitopes or the number of epitopes recognized and the severity of clinical reactions (Grade 0 vs. Grade I–IV in Api m 10 sensitized patients) [5] was found. There was also no discernable difference in Z‐scores. Controls (Api m 10‐negative sera from 2 HBV allergic patients (L7 and L9) and non‐allergic individuals (N1–N3) displayed no IgE‐recognition of Api m 10‐specific peptides (details not shown).

Signals measured in the array were calculated as Z‐scores of the Api m 10 peptides for Api m 10 isoform 1 and 2 based on the results obtained with 28 Api m 10‐reactive sera (Figure 2). Z‐scores are commonly used for comparability purposes between array detection signals, that is for normalization of signals between the various arrays that are probed with individual human serum antibodies and, thus, present individual signal characteristics due to differences in signals related to background and to specific peptides, respectively. Here, the Z‐score describes the measured peptide‐specific signal as a multiple of the standard deviation around the mean of the background signal. Therefore, the peptide‐specific signal is subtracted by the mean of the background‐specific signal of the array, and the signal difference subsequently divided by the standard deviation of the background signal that was obtained from technical replicates. More technical details and formulas are described elsewhere [25]. A calculated Z‐score larger than a cut‐off of two would indicate that the underlying measured signal of a specific peptide‐antibody binding relates to a true signal above background with a probability of more than 95%, assuming normal statistical distribution. A Z‐score larger than 3 would indicate a true signal above background with more than 99% probability. While a Z‐Score > 3 is often applied to identify specific binding to peptides, we first applied a Z‐Score > 2 that still includes a probability of 95% that a signal is above background for the numerical discrimination between positive and negative signals. Then we additionally applied a Z‐score of 10 or larger to strengthen the specificity of the epitope selection because of the high number of low binding sera with Z‐scores around 2 (Figure 2): Only Z‐scores at or above the cut‐off 2 were considered; the magnitude of Z‐scores was subdivided into steps of 10. The accumulated Z‐scores showed that the first two peptides P1 and P2, which exclusively contain the signal peptide sequence of Api m 10, were recognized by only five patient sera with very low Z‐scores.

**FIGURE 3:**
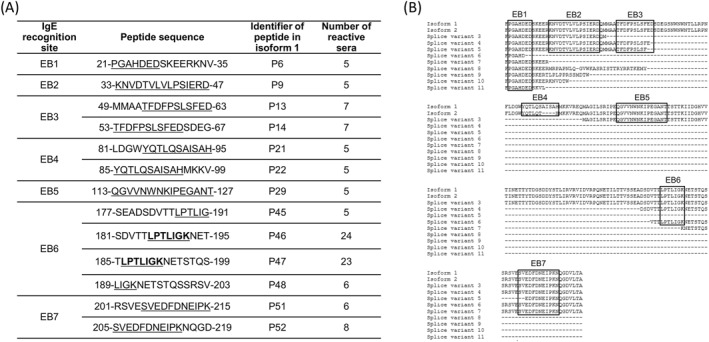
IgE‐reactive areas of Api m 10‐variants. (A) Seven IgE‐binding areas of Api m 10 isoform 1 were identified. Minimal putative peptide segments necessary for IgE‐binding are underlined. (B) Protein sequence alignment of all Api m 10‐variants with marked IgE‐binding regions (aligned with Kalign (2.0) (https://www.ebi.ac.uk/Tools/msa/kalign/)). The boxes mark the minimum IgE‐binding regions determined by peptide arrays with 28 Api m 10‐reactive sera.

In addition to a certain variability of individual sera, the peptide array results displayed characteristic recognition patterns with regard to the IgE‐binding of peptide regions. Using these recognition patterns, seven IgE‐binding regions were proposed for the Api m 10 isoform 1. As a criterion for such an IgE‐binding region, at least five allergic individuals showed IgE‐binding to its peptides with a Z‐score greater than 10. The number of at least 5 subjects has been chosen to comply with the minimal number of positive subjects as required by the IUIS allergen nomenclature (allergen.org) to register an allergen. We consider this approach also valid for the relevance of an epitope assignment. These criteria were in line with the selection of the most frequently and strongly bound peptides compared to measured background signals (Figure 3A). All identified IgE‐binding regions of the different Api m 10‐variants were plotted in a protein sequence alignment of the 11 putative Api m 10‐variants to show the identified preserved IgE‐binding motifs and regions (Figure 3B):

- The binding region EB1 consists of the peptide P6 (21‐PGAHDEDSKEERKNV‐35) and was recognized by five Api m 10‐positive sera. The peptides P69 of splice variant 6 (PFAHDVTTLPTLIGK), P97 of variant 7 (PGAHDEDSKEERKNE), P106 of variant 8 (PGAHDEDSKEERMRP), P115 of variant 9 (PGAHDEDSKERTLPL) and P120 of variant 11 (THSFPGAHDEDSKVL) showed a similar IgE recognition pattern (Table 2; Figure 3A,B). All these peptides share the sequence motif “PGAHDED”, which was therefore considered as the minimum binding region.
- Another IgE‐binding peptide that is in line with the above criteria is peptide P9 (33‐KNVDTVLVLPSIERD‐47). The whole peptide sequence of P9 is represented in isoforms 1 and 2 and splice variants 3 to 5, representing IgE‐binding area EB2 which was detected by 5 sera (Figure 3A,B).
- Peptide P13 (49‐MMAATFDFPSLSFED‐63) and P14 (53‐TFDFPSLSFEDSDEG‐67) were assigned to IgE‐detection area EB3, which was detected by seven Api m 10‐reactive sera. This area is represented in isoforms 1 and 2 and splice variant 4 and partly in splice variant 5, the common motif is “TFDFPSLSFED” (Figure 3A,B).
- The fourth binding region EB4, which contains peptides P21 (81‐LDGWYQQTLQSAISAH‐95) and P22 (85‐YQTLQSAISAHMKKV‐99), was recognized by five Api m 10‐reactive sera and is exclusively present in Api m 10‐isoforms 1 and 2.
- Peptide P29 (113‐QGVVNWNKIPEGANT‐127) displayed also a moderate IgE recognition sequence (EB5) recognized by five Api m 10‐reactive sera.
- For the peptides P46 (181‐SDVTTLPTLIGKNET‐195) and P47 (185‐TLPTLIGKNETSTQS‐199) of the IgE‐binding region EB6, the binding of 24 and respectively 23 patients' sera with a Z‐score higher than 10 was detected. The sequence thus represented a major recognition site and possibly an important epitope of Api m 10. This sequence region is also present in the IgE‐reactive peptides of splice variant 3, in which it extends to peptides P79, P80 and P81. Accordingly, the minimum recognition area was limited to the sequence motif “LPTLIGK”. This motif was also part of isoform specific peptides of splice variant 4 (P90, FEDSDVTTLPTLIGK) and 6 (P96, PGAHDVTTLPTLIGK), which were also recognized by the majority of the sera.
- EB7 was located at the C‐terminus of the protein and extended to peptides P51 (201‐RSVESVEDFDNEIPK‐215) and P52 (205‐SVEDFDNEIPKNQGD‐219). With recognition of 6–8 sera, it represented the second most frequently recognized region of Api m 10.

These IgE‐binding sites also represent the most frequently detected areas of isoform 2, splice variants 3, 4, 6 and 7. Splice variant 5 contains an area to which IgE antibodies of some allergic sera bound strongly. This extends to the variant specific peptides P91, P92 and P93 and may consist of the binding regions EB1 and EB7. For the splice variants 8, 10 and 11 no prominent recognition of specific peptides was observed. The peptides P116 and P117 of variant 9 may represent a further IgE‐detection area; both peptides are recognized by six (21.4%) of the 28 Api m 10‐sensitive sera (data not shown).

### 3.3 Biophysical Characterization of Recombinant Expressed Api m 10‐Variants

**FIGURE 4:**
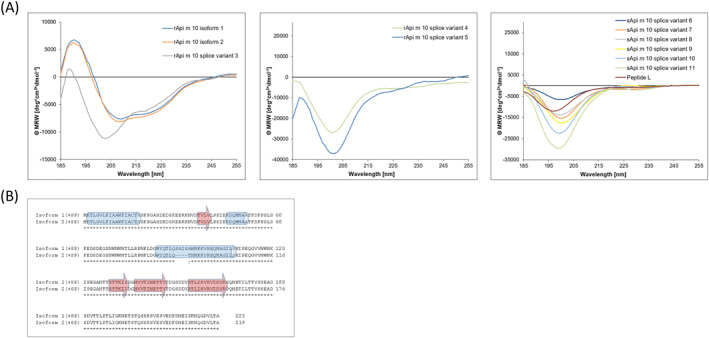
Circular dichroism spectroscopy analysis and secondary structure prediction of Api m 10‐variants 1–11 and peptide L. (A) The CD spectra of rApi m 10 isoforms and sApi m 10 splice variants are expressed as mean residue ellipticity Θ (*y*‐axis) at a given wavelength (*x*‐axis). (B) Secondary structure prediction of Api m 10‐variants. The sequences of Api m 10‐isoforms 1 and 2 were subjected to the secondary structure prediction tool *Quick2D toolkit* (https://toolkit.tuebingen.mpg.de/#/). α‐helices are shown as cylinders (blue), β‐sheets are depicted as block arrows (red).

The calculated molecular weights of the non‐glycosylated proteins expressed in *E. coli* were 22.86 and 22.53 kDa for rApi m 10 isoform 1 and 2, respectively, 16.69 kDa (splice variant 3), 9.75 kDa (splice variant 4) and 6.79 kDa (splice variant 5). The circular dichroism (CD) spectrum of rApi m 10 isoforms 1 and 2 showed a minimum at 209 nm, the rApi m 10 splice variant 3 showed a minimum at 204 nm, indicating that these recombinant proteins might contain β‐sheets, as well as α‐helices (Figure 4A). Secondary structure analysis using the program *Quick2D toolkit* (https://toolkit.tuebingen.mpg.de/#/) predicted three α‐helices and four β‐sheets (Figure 4B). These results are in concordance with recent findings [27]. Moreover, in its non‐mature state with the described signal peptide, an additional α‐helix (Api m 10 isoform (+signal peptide) 1 AS 1–18) and an additional β‐sheet (Api m 10 isoform 1 (+signal peptide) AS 37–41) were predicted. The CD spectra of rApi m 10 splice variants 4 and 5 showed no measurable formation of secondary structure elements (coiled‐coiled structure). Besides the recombinant variants 1 to 5 expressed in *E. coli*, the smaller splice variants 6 to 11 were purchased as synthetic peptides. The sApi m 10 splice variants 6 to 11 did not display any folding according to CD measurements (Figure 4A). For sApi m 10 splice variants 4 to 11, no discernable secondary structure elements could be predicted with *Quick2D toolkit*, since most of the areas were indicated as disordered. Dynamic light scattering (DLS) analysis revealed (according to distribution results) a hydrodynamic radius (R_H_) of 4.886 ± 0.752 nm for rApi m 10 isoform 1. R_H_ for rApi m 10 isoform 2 was 4.886 ± 0.913 nm. The R_H_ of peptide L was 1.340 ± 0.1177 nm. The large R_H_ values indicated the formation of aggregates for both isoforms and peptide L. According to mass distribution (> 99.8%), the measured particles were monodisperse. Further evidence for aggregation/dimerization of rApi m 10 isoforms 1 and 2 was obtained by analysis of MW in SDS‐PAGE under reducing conditions (23 kDa) (Supporting Information S1: Figure 3A) and non‐reducing conditions (45–55 kDa) (Supporting Information S1: Figure 3B).

### 3.4 Inhibition of IgE‐Binding With Immunodominant IgE‐Reactive Peptide (“Peptide L”)

**FIGURE 5:**
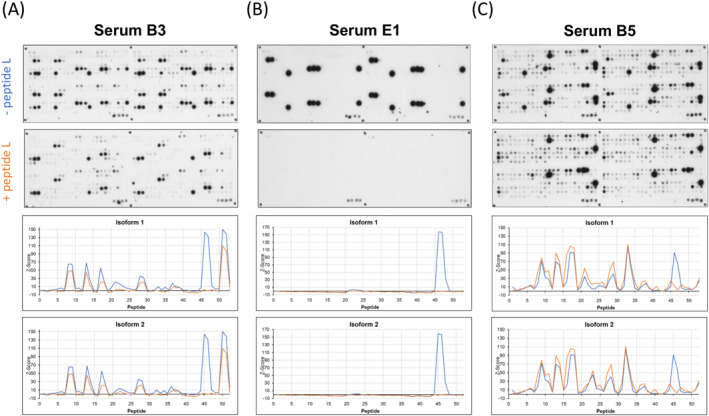
IgE‐inhibition of Api m 10‐peptides with peptide L. IgE‐binding of three human sera of Api m 10‐sensitized individuals preincubated with peptide L (second row) versus native (non‐preincubated) serum binding (first row) in Api m 10‐peptide arrays. Z‐score normalized data (for isoform 1 (third row) and isoform 2 (bottom row): blue curve: uninhibited; orange curve: inhibited with excess of peptide L (A) Serum B3, exposure time 1 min; (B) Serum E1, exposure time 15 s; (C) Serum B5, exposure time 30 s.

To verify the relevance of the identified IgE‐reactive sequence motif “LPTLIGK” from the major IgE‐binding region EB6, inhibition experiments with a synthetic peptide (referred to as “peptide L”) were performed. The peptide L contained the sequence of this motif twice, separated by a linker of glycines and serines (GS‐linker), resulting in the sequence: G**LPTLIGK**GSGGGGSG**LPTLIGK**G‐NH_2_. Figure 5 and Supporting Information S1: Figure 4 depict the IgE‐binding profiles of 5 Api m 10‐reactive sera. Peptide L specifically inhibits IgE‐binding to peptides that contain this motif: Using the sera B3, B5, E1, E3 and E8, the peptides P46 and P47 from isoform 1 and isoform 2, P79 to P81 from splice variant 3, P90 from splice variant 4 and P96 from splice variant 6 could be inhibited. Peptides without the specific sequence motif were not inhibited (Figure 5, Supporting Information S1: Figure 4).

### 3.5 Inhibition of IgE‐Binding to Peptides (On CelluSpot Slides) by Recombinant Api m 10

**FIGURE 6:**
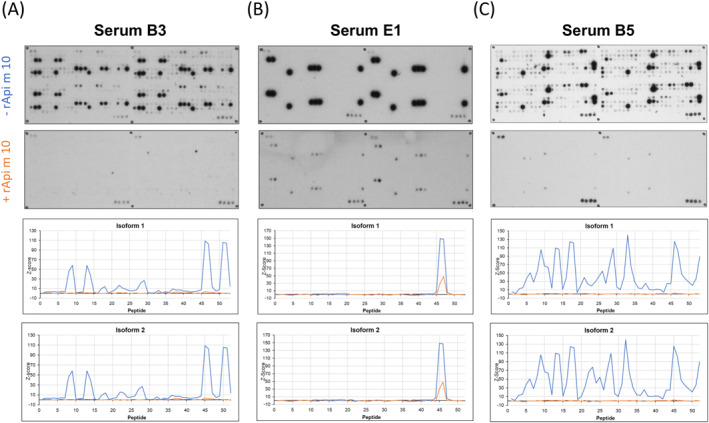
IgE‐inhibition of Api m 10‐peptides with recombinant Api m 10 isoform 1. Immunodetection of Api m 10‐peptide arrays (above) and Z‐score normalized data (below); blue curve: uninhibited; orange curve: inhibited with excess of rApi m 10 isoform 1. (A) Serum B3, exposure time 1 min; (B) Serum E1, exposure time 15 s; (C) Serum B5, exposure time 30 s.

Inhibition experiments with rApi m 10 were performed to determine whether the above described IgE‐binding regions (Figure 3A) are also accessible in the folded protein to IgE‐antibodies and to what extent the recognition of isoform‐specific peptides was based on cross‐reactivity. rApi m 10 isoform 1, including all sequences of the shorter variants gained from alternative splicing, was used to inhibit IgE‐binding of Api m 10‐reactive human sera to the peptides of the CelluSpot slides using the 5 sera B3, B5, E1 (Figure 6), and E3 and E8 (Supporting Information S1: Figure 5). Using the sera B3, B5 and E8, IgE‐binding to peptides was completely inhibited by rApi m 10 isoform 1, which indicates surface accessibility of the relevant peptide motifs. Further, the major linear peptide motif (peptide 46, 47) was almost completely inhibited in serum E1. Using serum E3, only minor IgE‐binding resided after inhibition with rApi m 10 isoform 1, which also indicated specificity of the identification of linear peptide motifs and their surface accessibility on the full‐length allergen.

### 3.6 The Major Linear Epitope Is Part of a Larger Repertoire of IgE‐Epitopes

**FIGURE 7:**
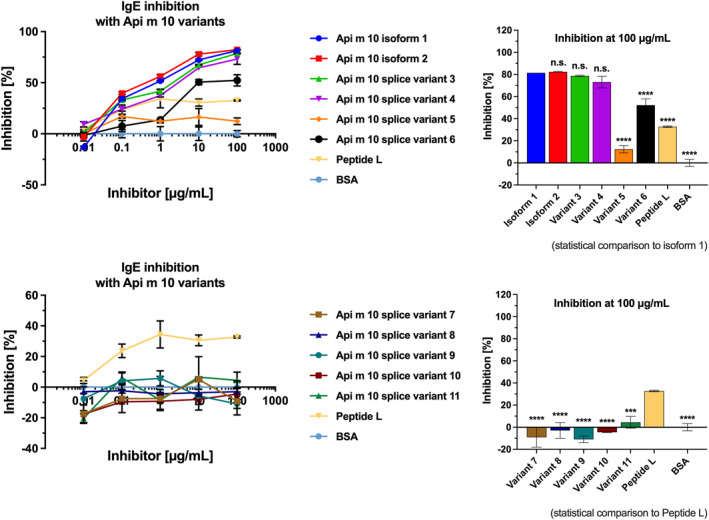
IgE‐inhibition ELISA. Dose‐dependent IgE‐inhibition was performed with a serum pool of five Api m 10‐reactive sera. Sera were incubated with different concentrations of rApi m 10 variants 1–11 and the peptide L. Inhibition with BSA was used as a negative control. Data are mean of two technical replicates ± standard deviations (SD). Statistical comparisons were performed by two‐way ANOVA adjusted for multiple comparisons according to Tukey. Statistical comparisons were performed with reference to either isoform 1 (upper panel) or peptide L (lower panel). For statistical significant results the following convention was used: ***p value < 0.001, and ****p value < 0.0001. The statistical analysis was performed with Graphpad Prism, version 10.6.0 for Macintosh.

ELISA inhibition experiments were performed with rApi m 10 isoform 1 coated onto microplate wells and using IgE from an Api m 10‐reactive HBV serum pool (Sera B3, B5, E1, E3 and E8). Here, with an inhibition between 81% and 52%, the purified Api m 10‐variants 1 and 2, and the splice variants 3, 4 and 6 reached similar levels of inhibition. The peptide L, consisting of two replicates of the identified highly IgE‐reactive peptide separated by a glycine‐serine‐linker reached an inhibition of 33%. This finding is in agreement with additional linear epitopes identified using IgE inhibition on peptide arrays (Figure 5 **,** Supporting Information S1: Figure 4). In contrast, Api m 10 variant 5 reached only 12% of IgE inhibition, indicating the importance of the highly IgE‐reactive sequence motif “LPTLIGK”, which is absent in Api m 10 variant 5. Api m 10‐variants 7–11 showed no significant inhibition in the inhibition ELISA (Figure 7). Accordingly, for an inhibitor concentration of 100 μg/mL Api m 10 isoform 1 induced significantly higher IgE inhibition than Api m 10‐variants 5 and 6, peptide L, and the BSA control while no significant differences were observed between inhibition induced by Api m 10 isoform 1 and isoform 2, as well as Api m 10‐variants 3 and 4 (Figure 7). Api m 10‐variants 7–11 all induced significant lower levels of IgE inhibition than peptide L (Figure 7).

### 3.7 Mouse IgG and Human IgE Recognize Both the Same Api m 10 Epitopes

After having identified seven IgE‐binding regions of human sera (EB1 to EB7) that were recognized by 5–24 human sera (Figure 3A,B), respectively, we investigated the epitope‐specific binding of murine IgG‐antibodies and compared it with the identified IgE‐binding epitopes of human antibodies. For this, binding regions of either female or male pooled mouse sera (each derived from five animals per sex) from rApi m 10‐immunized mice were investigated separately (Supporting Information S1: Figure 6). Whereas sera from both female and male mice detected EB2, EB6 and EB7 with high intensities (Z‐scores > 180 in males and > 100 in females), EB5 was recognized only with minor signals (Z‐score: 8 in males, 10 in females). Furthermore, another clear binding of sera from male mice that was slightly weaker in sera from female mice could be demonstrated at EB1 (Z‐score > 180 in males and 15 in mean in females). Interestingly, none of the pooled animal sera detected EB4. In comparison to human sera, mice sera (at least in pooled male sera) robustly detected one additional binding site (Z‐score: 75 in mean). The minimum recognition area represents “MAGILSRIP” that is located between EB4 and EB5 (Supporting Information S1: Figure 7).

## 4 Discussion

### 4.1 Epitope Mapping of Api m 10 and Its Variants via Peptide Microarray

The identification of B‐cell epitopes of allergenic proteins is an essential part of their characterization, which contributes to the understanding of the antigen‐antibody interaction of the human immune response. Peptide array technology offers a method for mapping linear epitopes and can be performed without the need for a purified antigen or antibody. Conformational IgE‐epitopes often contain AA segments which are present in the primary sequence of the allergen. Therefore, the mapping of linear peptides can potentially be used to identify putative stretches of conformational epitopes [28].

We performed peptide array experiments with a total of 28 Api m 10‐reactive sera and 5 controls (two sera from Api m 10‐negative HBV patients and three non‐allergic individuals) (Figures 1 and 2) to further elucidate IgE‐binding regions of Icarapin (Api m 10), one of the major allergens of HBV. IgE‐binding to different IgE recognition sites is individual for each patient's serum. Figure 2 summarizes the number of reactive sera for each peptide. From this figure, it can be deduced that the first two peptides, which exclusively contain the sequence of the signal peptide, were only recognized by a few sera and only with a low Z‐score. This might be either low affinity or non‐specific binding, as the signal peptide is most likely cleaved off in the mature form of Api m 10 and its variants and is not present in poison glands [18]. Apart from a certain variability of individual sera, several conserved recognition patterns of IgE‐binding of peptide regions were identified: Seven linear IgE‐binding regions could be identified for the Api m 10 isoform 1 (Figure 3A,B). These seven IgE‐binding regions covered almost all the IgE‐reactivities of the 28 patients sensitized to Api m 10. The sera B5 and E4 (ImmunoCAP class 4 to Api m 10) in addition to the listed putative IgE‐epitopes (Figure 3A,B), also recognized additional areas of the protein. This also correlates with the higher IgE titers, which showed a more sensitive detection of the peptide array. Whether these identified IgE‐binding regions also represent functional B cell epitopes of the protein is uncertain and requires further investigation.

The areas EB1, EB2 and EB5 (Figure 3A,B) comprise only one peptide each and are recognized by 18% of the Api m 10‐positive sera. In earlier studies on epitope mapping using peptide arrays, epitopes were defined as at least two overlapping IgE‐reactive peptides [31, 32]. This criterion is not the case for the above‐mentioned binding regions; however, some sera also recognized neighboring peptides (usually two peptides in total). The sequence which represents the IgE‐binding region EB6 can be interpreted as a major epitope of Api m 10. A major epitope is usually understood as a sequence area that contains at least two overlapping peptides and exhibits IgE‐reactivity with more than 50% of the patients' sera [27, 31, 33]. The two peptides of this epitope were recognized by 82% of the tested Api m 10‐sensitized sera with a Z‐score over 10 and even by 96% of the Api m 10‐positive sera with a Z‐score above 2. The semi‐quantitative nature of Z‐scores has been emphasized (see materials and methods). Based on the overlapping peptides of the peptide array, the minimal sequence of this epitope required to bind IgE‐antibodies could be set to a 7‐AA‐stretch with the sequence “LPTLIGK”.

Recently, a major IgE‐epitope of Api m 10 was identified in Api m 10 isoform 1 using a macroarray platform coated with 15‐mer peptides, with an overlap of 12 AA spanning the whole AA sequence of the mature protein. The peptide was described as Api m 10_160–174_ (AA sequence: ADSDVTTLPTLIGKN) [16], which resembles the immunodominant peptide LPTLIGK identified in this study. Rauber and coworkers identified three additional regions with IgE‐binding of more than 40% of the *n* = 40 tested Api m 10‐sensitized sera [16, 29]. However, the exact locations of the three additional IgE‐reactive regions on the Api m 10 sequence were not specified in more detail by Rauber and coworkers.

One limitation of our study is its relatively small cohort size (*n* = 28 patients), which limits the generalizability of the identified epitope profile to the entire population of HBV‐allergic patients, especially with respect to minor epitopes like EB4 and EB5 that were only recognized by a subset of patients.

### 4.2 Inhibition Experiments With Synthetic Peptide L and rApi m 10 Isoform 1 Showed a Significant Reduction of IgE‐Binding

To verify the relevance of the identified conserved strongly IgE‐reactive region EB6, inhibition experiments with a synthetic peptide, containing the identified predominant minimum recognition sequence LPTLIGK twice separated by a GS‐linker [33] and the rApi m 10 isoform 1 have been performed. The inhibition experiments using the synthetic peptide L (GLPTLIGKGSGGGGSGLPTLIGKG‐NH_2_) revealed that the short sequence motif “LPTLIGK” was sufficient to inhibit the IgE‐binding on the Api m 10 peptide microarray to the peptides P46 and P47 (Api m 10 isoform 1 and isoform 2), P79, P80 and P81 (Api m 10 splice variant 3), P90 (Api m 10 splice variant 4) and P96 (Api m 10 splice variant 6). The inhibition rates after pre‐incubation with the synthetic peptide L was 90% or even higher in five selected patients' sera, indicating that the minimum recognition sequence of EB6 is an immunodominant peptide in the IgE recognition of Api m 10 (Figure 5, Supporting Information S1: Figure 4). An average epitope contains 20 AA, of which only 2 to 5 are energetically critical for the protein‐protein interaction and represent the so‐called hot spot residues of the epitope [34]. Therefore, probably not all AA of a motif are essential for the recognition by the paratope of antibodies. A systematic exchange of one or several AA residues could be used to further characterize this epitope. By generating mutants with AA substitutions in this area, the influence of the epitope on the overall allergenicity of Api m 10 could also be elucidated.

The peptide microarrays inhibition experiments performed with full‐length Api m 10 isoform 1 showed significant, in some cases even complete, inhibition of IgE‐binding to the peptides of all isoforms (Figure 6, Supporting Information S1: Figure 5). This confirmed that the recognition of conserved linear epitopes of the variants was due to cross‐reactivity of IgE‐antibodies directed to isoform 1.

In ELISA inhibition experiments, the purified Api m 10‐isoforms 1 and 2, and the splice variants 3, 4 and 6 reached with between 81% and 52% similar levels of IgE‐inhibition. The peptide L, consisting of the two replicates of the identified highly IgE‐reactive peptide and a glycine‐serine‐linker in between reached an inhibition of 33%. In contrast, Api m 10 variant 5 reached only 12% of IgE inhibition, indicating the importance of the highly IgE‐reactive sequence motif “LPTLIGK”, which is absent in Api m 10 variant 5. Api m 10‐variants 7 to 11 showed no significant inhibition in the inhibition ELISA (Figure 7).

With regard to the highly IgE‐reactive sequence motif “LPTLIGK”, our results of the ELISA IgE‐immune inhibition analysis are in contrast to the findings of Rauber et al. where the major IgE‐epitope Api m 10_160–174_ achieved between 60% and 80% IgE inhibition in selected sera using the ImmunoCAP system [16, 29]. With 33% IgE‐inhibition potency in the pooled sera investigated in our study, the major linear IgE‐binding motif presents an IgE‐epitope or part of an IgE‐epitope with relevant IgE‐binding capacity, but does not fully cover the IgE‐epitope repertoire of Api m 10. Compared to 80% self‐inhibition using Api m 10, peptide L inhibited approximately half of the IgE‐binding capacity of Api m 10 in our ELISA inhibition experiment (Figure 7). Currently, the reason for these differences in the IgE inhibition potency of the respective linear motif in the two studies cannot be fully elucidated but can be most likely explained by different patient selection used in the two studies and using a serum pool versus selected individual sera. Furthermore, different experimental conditions of ELISA‐versus ImmunoCAP‐inhibition, and differences in assay sensitivity, respectively, may have played a role. However, both studies showed that the linear stretch cannot fully explain the IgE‐repertoire of full‐length Api m 10 in its folded form, suggesting that additional (most likely conformational) IgE‐epitopes play an important role in Api m 10‐sensitization.

One limitation of our study is the use of recombinant Api m 10 generated in *E. coli* lacking post‐translational glycosylation patterns normally present in HBV. Currently, natural, glycosylated Api m 10 is not commercially available, likely due to both its intrinsic instability and low frequency in HBV. Our choice of rApi m 10 generated in *E. coli* is supported by the results of Blank et al. that showed comparable IgE reactivity and capacity to activate basophils for both glycosylated Api m 10 (generated in HighFive insect cells) and non‐glycosylated Api m 10 (generated in *E. coli*) [11]. Therefore, the IgE reactivity of Api m 10 was suggested to be CCD‐independent [11]. However, it currently remains unknown how the lack of glycosylation affects the proteins' structure, stability, and overall allergenicity.

It is unlikely that this lack of glycosylation explains 50% of IgE‐binding to Api m 10 not being inhibited by the linear peptide L, as higher percentages of IgE‐binding inhibition were observed for the also non‐glycosylated, full‐length Api m 10 isoform 1 generated in *E. coli*. Most likely, the difference in IgE‐binding can be attributed to conformational epitopes which are present in the full‐length Api m 10, but not peptide L.

Conclusively, investigating the contribution of Api m 10 glycosylation to protein structure, stability, and overall allergenicity would require direct comparison of our recombinant Api m 10 generated in *E. coli* to natural Api m 10 isolated from HBV. However, this important and interesting question can only be addressed upon availability of sufficient amounts of natural Api m 10.

### 4.3 Similar Epitope Recognition of Man and Mouse

Concerning the observed cross‐species conservation of immunodominance it appears noteworthy to clarify how this model (‐in which immunization was performed with full‐length rApi m 10‐) reflects natural sensitization in humans. In contrast to natural (*n*) Api m 10, which is commercially unavailable, rApi m 10 expressed in *E. coli* does not contain posttranslationally added cross‐reactive carbohydrate determinants (CCDs) [11]. This lack of glycosylation leads to different molecular weights (35 kDa for rApi m 10; 55 kDa for nApi m 10). However, findings obtained with the non‐glycosylated recombinant protein expressed in *E. coli* have previously been shown to match those with the glycosylated protein [11]. In line with this, we could demonstrate in a previous study, that preincubation of rApi m 10‐coated ELISA plates with pooled sera from rApi m 10‐immunized mice significantly inhibited Api m 10‐specific binding of human IgE in a dose‐dependent manner [35] pointing toward epitope similarity. Some differences in function and expression of human versus mouse humoral immune responses were reported in the past [36], that need to be taken into account when using mice as preclinical models of human disease. In addition to differences in FcR expression there are well‐known differences in expression of Ig isotypes between mice and humans, and direct correlations between subtypes within classes in each species are hard to make [36]. Whereas both, human and mice produce IgM, IgE, IgD, and IgA (in humans two subtypes (IgA1 and IgA2)), the subtypes of IgG vary between both species: Mice produce IgG1, IgG2a, IgG2b, and IgG3 and humans IgG1, IgG2, IgG3, and IgG4 [36]. With regard to potential differences in antigen recognition, we compared the binding regions of Api m 10‐specific antibodies from both species. We could show that IgG‐antibodies from mice and IgE‐antibodies from humans both robustly bind to seven different protein sequences, with six of them being identical between both species. Thus, mouse and human antibodies recognize the same Api m 10 epitopes. To our knowledge this is the first time that this cross‐species epitope similarity has been shown for a HBV allergen. However, the finding is in line with previous reports from Vrtala et al. and Focke et al. who demonstrated for grass pollen allergens and dog albumin, respectively, that immunodominant human IgE epitopes were similarly recognized by murine IgG antibodies induced by recombinant allergen fragments [37] or synthetic peptides [38]. To this point there has not been direct evidence that human and mouse antibodies recognize identical epitopes on the honey bee venom allergen Api m 10. Most published research focuses on human IgE reactivity. Api m 10 itself is classified as a “conserved protein,” with homologs—known as icarapin‐like proteins—identified on the DNA level in a broad range of insects, including other bees, bumblebees, wasps, ants, beetles, flies, mosquitoes, and more [27]. However, while Api m 10 is conserved as a whole, the conservation of specific epitopes recognized by different species has not been reported before. Usually, recognition of identical epitopes between different species also suggests a high degree of conservation of the respective protein. This is usually coupled with a distinct protein folding recognized by the respective antibodies. However, in line with our experimental results and previous secondary structure prediction [27] the Api m 10‐isoforms 1 and 2 only display isolated secondary structure elements and the Api m 10 molecule appears to be largely unfolded with the potential existence of different tertiary structures and extensive changes in protein folding upon binding of a potential ligand [27]. Therefore, a highly conserved, but so far unrecognized folding of Api m 10 is unlikely to be the reason of the conserved epitope recognition between men and mice. In contrast, we hypothesize that similarities in Api m 10 processing between both species might contribute to the conserved antibody responses. Immunologically, the induction of antigen‐specific B cell responses requires CD4 T cell‐driven B cell activation. However, while B cells usually recognize the intact and folded antigen with their B cell receptor, T cells can only recognize antigen‐derived peptides presented on MHC molecules of Antigen‐presenting cells. These peptides are generated in the endolysosomes of Antigen‐presenting cells using a mixture of acid proteases. While there are species‐specific differences in these proteases, many are highly conserved between species [40, 41, 42]. Therefore, we assume that the conservation of these proteases between both species results in a very similar set of peptides being generated from Api m 10 in men and mice. This set of peptides determines the specificity of the activated Api m 10‐specific T cells which in turn authorize B cells of the same specificity, resulting in the observed, highly conserved Api m 10‐specific B cell responses. While at the time given this is a hypothesis that requires confirmation in independent studies, our results demonstrate that BALB/c mice represent an appropriate model to study Api m 10‐specific immune responses. Although the results from mouse models cannot be directly transferred to humans, our result of Api m 10 epitopes identically recognized by mice and humans supports the scientific importance of the information obtained in preclinical models in BALB/c mice.

### 4.4 Characterization of Secondary Structure of Api m 10‐Variants

rApi m 10 isoforms 1 and 2 as well as rApi m 10 splice variants 3–5 were produced as recombinant proteins in *E. coli,* sApi m 10 splice variants 6–11 as well as the peptide L were used as synthetic peptides. Secondary structure prediction from the rApi m 10 variants using the *Quick2D toolkit* (https://toolkit.tuebingen.mpg.de/#/) indicated no folding units for the C‐terminal region of the protein (Figure 4B). In an additional analysis of the protein for disordered regions using the *GeneSilico MetaDisorder Tool* (http://iimcb.genesilico.pl/metadisorder), the C‐terminal region was classified as unstructured. Hence, the identified major epitope EB6 of Api m 10 appears to be in an unstructured region. A comparison with the previously published major IgE‐epitope of Api m 10, indicated that their identified major Api m 10‐epitope was also located within the same unstructured area [27]. This location in mainly unstructured areas may have eased the efficient characterization of the identified epitopes by peptide array experiments. We hypothesize that the other identified IgE‐binding regions of Api m 10 might represent parts of discontinuous epitopes. For example, the IgE‐binding region EB2 is located in the first predicted β‐sheet of Api m 10 and could form an epitope with its adjacent β‐sheets (Figure 4B). In addition, the IgE‐binding region EB4 lies in the second predicted α‐helix, which could represent a simple conformational epitope or correspond to part of an epitope formed by the folding of the protein. To confirm these hypotheses, a determination of the spatial structure of the allergen, for example by NMR analysis or X‐ray structure determination, is required. These structural determinations could specifically confirm or refute the hypothesis of discontinuous epitopes (e.g., by revealing the 3D proximity of identified linear segments).

Our DLS measurements and molecular mass determination by SDS‐PAGE and native PAGE have revealed that rApi m 10 isoforms 1 and 2 are present in a dimeric form. As a consequence, repetitive presentation of major epitopes, such as the motif “LPTLIGK”, due to aggregated structures may contribute, at least in part, to the allergenicity of Api m 10 as a major HBV allergen. However, at this point this hypothesis remains to be experimentally demonstrated in functional effector cell assays, such as basophil or mast cell activation tests or mediator release assays. Alternative causes for aggregation could lie in the recombinant production of Api m 10, for example, misfolding of a small portion of the protein or expression system artifacts, which we cannot rule out completely.

## 5 Conclusion and Outlook

The structural, clinical and preclinical insights into Api m 10 and its immunodominant epitopes gained in this study provide substantial insights for the future development of active and passive VIT as well as further treatment approaches [43, 44]. Increasing evidence from clinical and preclinical studies [11, 12, 13, 14, 15, 45] indicate that Api m 10 amounts in complex HBV VIT‐products are low and might not suffice to induce robust protective antibody responses. The underrepresentation of Api m 10 in bee venom therapy products and its potential use as a predictor of treatment failure in sensitized subjects calls for prospective clinical studies with focus on this significant limitation. Treatment strategies/HBV VIT‐products with higher amounts of Api m 10 seem feasible, which would require novel product development.

The results of the epitope mapping of Api m 10 and identification of immunodominant peptides may support the concept to rationally design therapeutic preparations based on relevant allergen components [45] and their epitopes, for example, by (i) enrichment strategies of labile native allergens such as Icarapin during manufacture of allergen immunotherapy (AIT) products or (ii) by spiking of HBV with Api m 10 produced by recombinant DNA technology or its immunodominant peptides or (iii) by removing the trigger by blocking the allergen (e.g., applying nanobody technology) or (iv) by inactivation the IgE/FcεRI complex (e.g., by introducing novel ligands as disruptors) [43, 44, 47, 48]. For the identification of epitopes, we applied a rather strict cut‐off value of Z‐scores ≥ 10, instead of *Z* > 2 or *Z* > 3. Our rational was to strengthen the probability of true positive signals while accepting the risk that some epitopes between *Z* > 2 and 10 might have been missed. Thus, future work should verify the biological relevance of our findings at the Api m 10 protein level.

To date, spiking allergen extracts with recombinant allergens has successfully been applied in vitro routine diagnostics to increase sensitivity [48], but not in AIT‐products. Therapeutic approaches based on recombinant DNA technology so far have been tested in clinical trials only (reviewed in [49]) and no AIT‐product based on recombinant DNA technology has obtained marketing authorization, yet. An added value of these recombinant drug products based on a single allergen only could not be demonstrated in comparison to established extract‐based AIT [49]. By using purified allergens or peptides the spectrum of allergens is reduced, but in contrast to extracts from natural allergen sources the allergens can be chosen according to their importance and included in a dose that is adequate to achieve the desired clinical improvement [50].

It is noteworthy to mention, that at the time given in the European Union, recombinant AIT products, in contrast to synthetic or extract‐based allergen products, must be authorized in accordance with the Annex to Regulation (EC) 726/2004 via a centralized procedure, as this is mandatory for all medicinal products manufactured using recombinant DNA in biotechnological processes [51]. In contrast to well‐stablished molecular in vitro diagnostics for Hymenoptera venom allergy [52], immunotherapy tailored to the individual patient has been difficult to implement within regulatory guidelines to date, as no patient‐specific component‐resolved personalized formulations, but only finished medicinal products with a defined composition, are suitable for approval [49]. This could change in the future in line with current developments in personalized approaches to the treatment of cancer [53]. The ongoing reform of the pharmaceutical legislation, which has been proposed by the European Commission (EC) in a draft Directive and Regulation on 26th of April 2023 [54], may facilitate a personalized approach since it foresees the possibility of a Regulatory Sandbox^1^ as initiative to support innovative developments where there are challenges within the scope of regulations.

## Author Contributions

**Kathrin Elisabeth Paulus‐Tremel:** conceptualization, methodology, data curation, investigation, validation, formal analysis, supervision, visualization, project administration, writing – original draft, writing – review and editing. **Michelle Beatrice Wolff:** data curation, investigation, validation, formal analysis, visualization, writing – review and editing. **Natalija Novak:** data curation, writing – review and editing, formal analysis, validation. **Nicola Wagner:** data curation, formal analysis, writing – review and editing, validation. **Alisa Landgraf:** data curation, investigation, validation, formal analysis, writing – original draft, writing – review and editing, methodology, software, project administration. **Stefan Schülke** validation, formal analysis, data curation, writing – review and editing, visualization. **Thomas Holzhauser:** methodology, data curation, formal analysis, supervision, validation, writing – original draft, writing – review and editing. **Vera Mahler:** conceptualization, methodology, data curation, software, validation, formal analysis, supervision, resources, project administration, writing – original draft, writing – review and editing.

## Funding

The authors have nothing to report.

## Disclosure

The views expressed in this manuscript are the personal views of the authors and may not be understood or quoted as being made on behalf of or reflecting the position of the respective national competent authority, the European Medicines Agency, or one of its committees or working parties.

AL: “The contribution to the present work was performed in (partial) fulfillment of the requirements for obtaining the degree “Dr. rer. biol. hum.” at the Friedrich‐Alexander‐Universität Erlangen‐Nürnberg (FAU).”

## Conflicts of Interest

N.N. declares grants or contracts from Alk Abello, consulting fees, payment or honoraria for lectures, presentations, speakers bureaus, manuscript writing or educational events, payment for expert testimony, support for attending meetings and/or trave, participation on a Data Safety Monitoring Board or Advisory Board l from Abbvie, Alk Abello, Almirall, Bencard Allergy Therapeutics, Blueprint, Biogen, Bristol‐Myers Squibb, DocCheck, Eli Lilly, Galderma, HAL Allergie, lncyte, Leo Pharma, Janssen, Leti Pharma, Lofarma, Moonlake, Novartis, Phadia, Regenoron, Sanofi Genzyme, Stallergenes Greer, Streamed up, Thermo Fisher Scientific, UCB. All remaining authors declare that the research was conducted in the absence of any commercial or financial relationships that could be construed as a potential conflict of interest.

## Supporting information


Supporting Information S1

## Data Availability

The data that support the findings of this study are available on request from the corresponding author. The data are not publicly available due to privacy or ethical restrictions.

## Endnotes

^1^
A regulatory sandbox may be understood as a time‐limited regulatory framework that allows the testing of a product or approaches in a real‐world environment under regulatory supervision to facilitate the development and authorization of innovative products. The legal basis of a regulatory sandbox is a so‐called “experimentation clause”, that allows exemptions from existing requirements, in order to try out novel regulatory solutions.
