# Supplementary material for: Insights into Api m 10‐Isoforms and Splice Variants: More Than One Major IgE‐Binding Epitope
Source: Clin Transl Allergy. 2026 Mar 6;16(3):e70151. doi: 10.1002/clt2.70151 (PMC12965849; doi:10.1002/clt2.70151)
Supplement: Supplementary file 1 — Supporting Information S1 [file CLT2-16-e70151-s001.pdf]

Online-Supplement: Insights into Api m 10 isoforms and splice variants: More than one major IgE-binding epitope

Kathrin Elisabeth Paulus-Tremel[1,2†], Michelle Beatrice Wolff[1†], Natalija Novak[3], Nicola Wagner[4], Alisa Landgraf[1,5], Stefan Schülke[1], Thomas Holzhauser[1], Vera Mahler[1,4,5*]

[1]*Division Allergology, Paul-Ehrlich-Institut, Langen, Germany*
[2]*present address: Faculty of Life Sciences: Food, Nutrition and Health, University Bayreuth, Kulmbach, Germany*
[3]*Centre for Skin Diseases, Department of Dermatology and Allergy, University Hospital Bonn, Bonn, Germany*
[4]*Department of Dermatology, University Hospital Erlangen, Erlangen, Germany*
[5]*Friedrich-Alexander-Universität Erlangen-Nürnberg (FAU), Germany*

[†]These authors share first authorship.

*** Correspondence:** Prof. Vera Mahler, MD

Paul-Ehrlich-Institut, Division Allergology,
Paul-Ehrlich-Str. 51-59,
D-62335 Langen, Germany;
phone: +49 6103 77 2400 Fax: +49 6103 77 1258;
Email: vera.mahler@pei.de

Acknowledgements

We thank Stefanie Randow, Ann-Christine Junker, Elke Völker, Marcel Schöne and Meike Arend for excellent technical assistance.

Disclaimer

The views expressed in this manuscript are the personal views of the authors and may not be understood or quoted as being made on behalf of or reflecting the position of the respective national competent authority, the European Medicines Agency, or one of its committees or working parties.

AL: “The contribution to the present work was performed in (partial) fulfillment of the requirements for obtaining the degree “Dr. rer. biol. hum.” at the Friedrich-Alexander-Universität Erlangen-Nürnberg (FAU).”

	1	2	3	4	5	6	7	8	9	10	11	12	13	14	15	16	17	18	19	20	21	22	23	24
A	Biotin Control E6 1:3000	Biotin Control E6 1:3000	DMSO	DMSO	Api m 10 V1 P1 Box 4 A1	Api m 10 V1 P2 Box 4 A2	Api m 10 V1 P3 Box 4 A3	Api m 10 V1 P4 Box 4 A4	Api m 10 V1 P5 Box 4 A5	Api m 10 V1 P6 Box 4 A6	Api m 10 V1 P7 Box 4 A7	Api m 10 V1 P8 Box 4 A8	Api m 10 V1 P9 Box 4 A9	Api m 10 V1 P10 Box 4 A10	Api m 10 V1 P11 Box 4 A11	Api m 10 V1 P12 Box 4 A12	Api m 10 V1 P13 Box 4 B1	Api m 10 V1 P14 Box 4 B2	Api m 10 V1 P15 Box 4 B3	Api m 10 V1 P16 Box 4 B4	Api m 10 V1 P17 Box 4 B5	Api m 10 V1 P18 Box 4 B6	Api m 10 V1 P19 Box 4 B7	Api m 10 V1 P20 Box 4 B8
B	Api m 10 V1 P21 Box 4 B9	Api m 10 V1 P22 Box 4 B10	Api m 10 V1 P23 Box 4 B11	Api m 10 V1 P24 Box 4 B12	Api m 10 V1 P25 Box 4 C1	Api m 10 V1 P26 Box 4 C2	Api m 10 V1 P27 Box 4 C3	Api m 10 V1 P28 Box 4 C4	Api m 10 V1 P29 Box 4 C5	Api m 10 V1 P30 Box 4 C6	Api m 10 V1 P31 Box 4 C7	Api m 10 V1 P32 Box 4 C8	Api m 10 V1 P33 Box 4 C9	Api m 10 V1 P34 Box 4 C10	Api m 10 V1 P35 Box 4 C11	Api m 10 V1 P36 Box 4 C12	Api m 10 V1 P37 Box 4 D1	Api m 10 V1 P38 Box 4 D2	Api m 10 V1 P39 Box 4 D3	Api m 10 V1 P40 Box 4 D4	Api m 10 V1 P41 Box 4 D5	Api m 10 V1 P42 Box 4 D6	Api m 10 V1 P43 Box 4 D7	Api m 10 V1 P44 Box 4 D8
C	Api m 10 V1 P45 Box 4 D9	Api m 10 V1 P46 Box 4 D10	Api m 10 V1 P47 Box 4 D11	Api m 10 V1 P48 Box 4 D12	Api m 10 V1 P49 Box 4 E1	Api m 10 V1 P50 Box 4 E2	Api m 10 V1 P51 Box 4 E3	Api m 10 V1 P52 Box 4 E4	Api m 10 V1 P53 Box 4 E5	DMSO	DMSO	DMSO	DMSO	DMSO	DMSO	DMSO	DMSO	DMSO	DMSO	DMSO	DMSO	DMSO	DMSO	DMSO
D	DMSO	DMSO	DMSO	DMSO	DMSO	DMSO	DMSO	DMSO	DMSO	Api m 10 V2 P54 Box 8 A6	Api m 10 V2 P55 Box 8 A7	Api m 10 V2 P56 Box 8 A8	Api m 10 V2 P57 Box 8 A9	DMSO	Api m 10 V3 P58 Box 8 A10	Api m 10 V3 P59 Box 8 A11	Api m 10 V3 P60 Box 8 A12	Api m 10 V3 P61 Box 8 B1	Api m 10 V3 P62 Box 8 B2	Api m 10 V3 P63 Box 8 B3	Api m 10 V3 P64 Box 8 B4	Api m 10 V3 P65 Box 8 B5	Api m 10 V3 P66 Box 8 B6	Api m 10 V3 P67 Box 8 B7
E	Api m 10 V3 P68 Box 8 B8	Api m 10 V3 P69 Box 8 B9	Api m 10 V3 P70 Box 8 B10	Api m 10 V3 P71 Box 8 B11	Api m 10 V3 P72 Box 8 B12	Api m 10 V3 P73 Box 8 C1	Api m 10 V3 P74 Box 8 C2	Api m 10 V3 P75 Box 8 C3	Api m 10 V3 P76 Box 8 C4	Api m 10 V3 P77 Box 8 C5	Api m 10 V3 P78 Box 8 C6	Api m 10 V3 P79 Box 8 C7	Api m 10 V3 P80 Box 8 C8	Api m 10 V3 P81 Box 8 C9	Api m 10 V3 P82 Box 8 C10	Api m 10 V3 P83 Box 8 C11	Api m 10 V3 P84 Box 8 C12	Api m 10 V3 P85 Box 8 D1	Api m 10 V3 P86 Box 8 D2	Api m 10 V3 P87 Box 8 D3	DMSO	Api m 10 V4 P88 Box 8 D4	Api m 10 V4 P89 Box 8 D5	Api m 10 V4 P90 Box 8 D6
F	Api m 10 V5 P91 Box 8 D7	Api m 10 V5 P92 Box 8 D8	Api m 10 V5 P93 Box 8 D9	DMSO	Api m 10 V6 P94 Box 8 D10	Api m 10 V6 P95 Box 8 D11	Api m 10 V6 P96 Box 8 D12	DMSO	Api m 10 V7 P97 Box 8 E1	Api m 10 V7 P98 Box 8 E2	Api m 10 V7 P99 Box 8 E3	Api m 10 V7 P100 Box 8 E4	Api m 10 V7 P101 Box 8 E5	Api m 10 V7 P102 Box 8 E6	Api m 10 V7 P103 Box 8 E7	Api m 10 V7 P104 Box 8 E8	Api m 10 V7 P105 Box 8 E9	DMSO	Api m 10 V8 P106 Box 8 E10	Api m 10 V8 P107 Box 8 E11	Api m 10 V8 P108 Box 8 E12	Api m 10 V8 P109 Box 8 F1	Api m 10 V8 P110 Box 8 F2	Api m 10 V8 P111 Box 8 F3
G	Api m 10 V8 P112 Box 8 F4	Api m 10 V8 P113 Box 8 F5	DMSO	Api m 10 V9 P114 Box 8 F6	Api m 10 V9 P115 Box 8 F7	Api m 10 V9 P116 Box 8 F8	Api m 10 V9 P117 Box 8 F9	Api m 10 V9 P118 Box 8 F10	DMSO	Api m 10 V 10 P119 Box 8 F11	DMSO	Api m 10 V 11 P120 Box 8 F12	DMSO	DMSO	DMSO	DMSO	DMSO	DMSO	DMSO	DMSO	DMSO	DMSO	DMSO	DMSO
H	DMSO	DMSO	DMSO	DMSO	DMSO	DMSO	DMSO	DMSO	DMSO	DMSO	DMSO	DMSO	DMSO	DMSO	DMSO	DMSO	DMSO	DMSO	DMSO	DMSO	DMSO	DMSO	DMSO	DMSO
I	DMSO	DMSO	DMSO	DMSO	Api m 10 V1 P1 Box 4 A1	Api m 10 V1 P2 Box 4 A2	Api m 10 V1 P3 Box 4 A3	Api m 10 V1 P4 Box 4 A4	Api m 10 V1 P5 Box 4 A5	Api m 10 V1 P6 Box 4 A6	Api m 10 V1 P7 Box 4 A7	Api m 10 V1 P8 Box 4 A8	Api m 10 V1 P9 Box 4 A9	Api m 10 V1 P10 Box 4 A10	Api m 10 V1 P11 Box 4 A11	Api m 10 V1 P12 Box 4 A12	Api m 10 V1 P13 Box 4 B1	Api m 10 V1 P14 Box 4 B2	Api m 10 V1 P15 Box 4 B3	Api m 10 V1 P16 Box 4 B4	Api m 10 V1 P17 Box 4 B5	Api m 10 V1 P18 Box 4 B6	Api m 10 V1 P19 Box 4 B7	Api m 10 V1 P20 Box 4 B8
J	Api m 10 V1 P21 Box 4 B9	Api m 10 V1 P22 Box 4 B10	Api m 10 V1 P23 Box 4 B11	Api m 10 V1 P24 Box 4 B12	Api m 10 V1 P25 Box 4 C1	Api m 10 V1 P26 Box 4 C2	Api m 10 V1 P27 Box 4 C3	Api m 10 V1 P28 Box 4 C4	Api m 10 V1 P29 Box 4 C5	Api m 10 V1 P30 Box 4 C6	Api m 10 V1 P31 Box 4 C7	Api m 10 V1 P32 Box 4 C8	Api m 10 V1 P33 Box 4 C9	Api m 10 V1 P34 Box 4 C10	Api m 10 V1 P35 Box 4 C11	Api m 10 V1 P36 Box 4 C12	Api m 10 V1 P37 Box 4 D1	Api m 10 V1 P38 Box 4 D2	Api m 10 V1 P39 Box 4 D3	Api m 10 V1 P40 Box 4 D4	Api m 10 V1 P41 Box 4 D5	Api m 10 V1 P42 Box 4 D6	Api m 10 V1 P43 Box 4 D7	Api m 10 V1 P44 Box 4 D8
K	Api m 10 V1 P45 Box 4 D9	Api m 10 V1 P46 Box 4 D10	Api m 10 V1 P47 Box 4 D11	Api m 10 V1 P48 Box 4 D12	Api m 10 V1 P49 Box 4 E1	Api m 10 V1 P50 Box 4 E2	Api m 10 V1 P51 Box 4 E3	Api m 10 V1 P52 Box 4 E4	Api m 10 V1 P53 Box 4 E5	DMSO	DMSO	DMSO	DMSO	DMSO	DMSO	DMSO	DMSO	DMSO	DMSO	DMSO	DMSO	DMSO	DMSO	DMSO
L	DMSO	DMSO	DMSO	DMSO	DMSO	DMSO	DMSO	DMSO	DMSO	Api m 10 V2 P54 Box 8 A6	Api m 10 V2 P55 Box 8 A7	Api m 10 V2 P56 Box 8 A8	Api m 10 V2 P57 Box 8 A9	DMSO	Api m 10 V3 P58 Box 8 A10	Api m 10 V3 P59 Box 8 A11	Api m 10 V3 P60 Box 8 A12	Api m 10 V3 P61 Box 8 B1	Api m 10 V3 P62 Box 8 B2	Api m 10 V3 P63 Box 8 B3	Api m 10 V3 P64 Box 8 B4	Api m 10 V3 P65 Box 8 B5	Api m 10 V3 P66 Box 8 B6	Api m 10 V3 P67 Box 8 B7
M	Api m 10 V3 P68 Box 8 B8	Api m 10 V3 P69 Box 8 B9	Api m 10 V3 P70 Box 8 B10	Api m 10 V3 P71 Box 8 B11	Api m 10 V3 P72 Box 8 B12	Api m 10 V3 P73 Box 8 C1	Api m 10 V3 P74 Box 8 C2	Api m 10 V3 P75 Box 8 C3	Api m 10 V3 P76 Box 8 C4	Api m 10 V3 P77 Box 8 C5	Api m 10 V3 P78 Box 8 C6	Api m 10 V3 P79 Box 8 C7	Api m 10 V3 P80 Box 8 C8	Api m 10 V3 P81 Box 8 C9	Api m 10 V3 P82 Box 8 C10	Api m 10 V3 P83 Box 8 C11	Api m 10 V3 P84 Box 8 C12	Api m 10 V3 P85 Box 8 D1	Api m 10 V3 P86 Box 8 D2	Api m 10 V3 P87 Box 8 D3	DMSO	Api m 10 V4 P88 Box 8 D4	Api m 10 V4 P89 Box 8 D5	Api m 10 V4 P90 Box 8 D6
N	Api m 10 V5 P91 Box 8 D7	Api m 10 V5 P92 Box 8 D8	Api m 10 V5 P93 Box 8 D9	DMSO	Api m 10 V6 P94 Box 8 D10	Api m 10 V6 P95 Box 8 D11	Api m 10 V6 P96 Box 8 D12	DMSO	Api m 10 V7 P97 Box 8 E1	Api m 10 V7 P98 Box 8 E2	Api m 10 V7 P99 Box 8 E3	Api m 10 V7 P100 Box 8 E4	Api m 10 V7 P101 Box 8 E5	Api m 10 V7 P102 Box 8 E6	Api m 10 V7 P103 Box 8 E7	Api m 10 V7 P104 Box 8 E8	Api m 10 V7 P105 Box 8 E9	DMSO	Api m 10 V8 P106 Box 8 E10	Api m 10 V8 P107 Box 8 E11	Api m 10 V8 P108 Box 8 E12	Api m 10 V8 P109 Box 8 F1	Api m 10 V8 P110 Box 8 F2	Api m 10 V8 P111 Box 8 F3
O	Api m 10 V8 P112 Box 8 F4	Api m 10 V8 P113 Box 8 F5	DMSO	Api m 10 V9 P114 Box 8 F6	Api m 10 V9 P115 Box 8 F7	Api m 10 V9 P116 Box 8 F8	Api m 10 V9 P117 Box 8 F9	Api m 10 V9 P118 Box 8 F10	DMSO	Api m 10 V 10 P119 Box 8 F11	DMSO	Api m 10 V 11 P120 Box 8 F12	DMSO	DMSO	DMSO	DMSO	DMSO	DMSO	DMSO	DMSO	DMSO	DMSO	DMSO	DMSO
P	DMSO	DMSO	DMSO	DMSO	DMSO	DMSO	DMSO	DMSO	DMSO	DMSO	DMSO	DMSO	DMSO	DMSO	DMSO	DMSO	DMSO	DMSO	DMSO	DMSO	Biotin Control E6 1:1250	Biotin Control E6 1:1250	Biotin Control E6 1:1250	Biotin Control E6 1:1250

Supplemental Figure 1: Spotting-Layout of Api m 10 peptide microarray. The different isoforms were highlighted in color. In addition to the peptides listed in **Table 2**, biotinylated control peptides ("Biotin Control") were spotted on the cellulose membrane. DMSO was applied to positions that did not contain peptides. The illustrated layout was spotted in two identical segments (2x 384 spots) left and right on the cellulose carrier

(A)

Serum B3

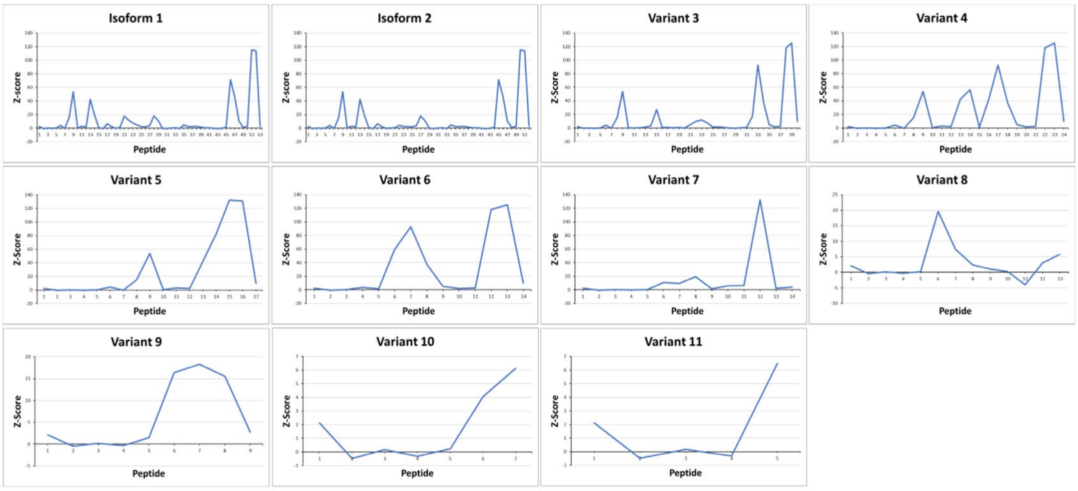

(B)

Serum E1

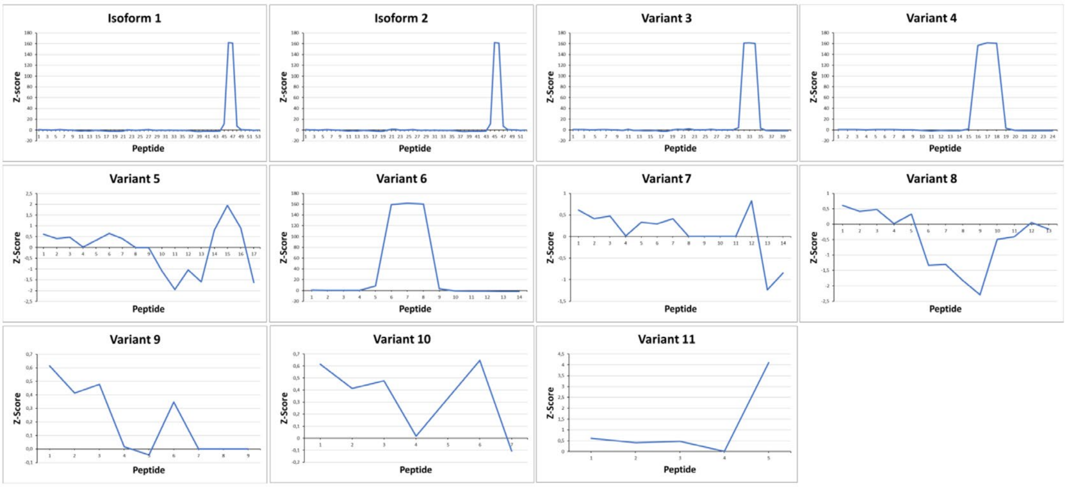

(C)

Serum B5

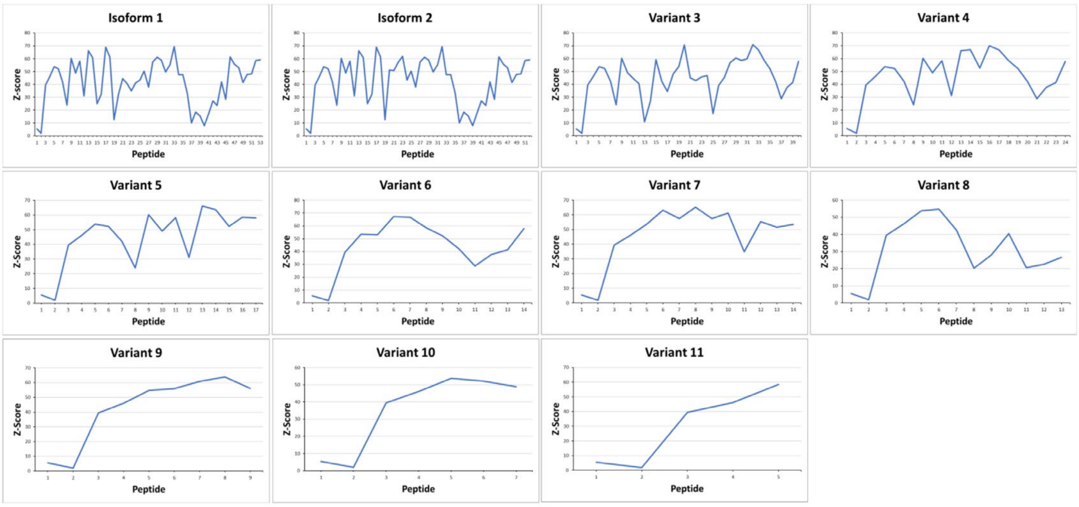

Supplemental Figure 2: IgE recognition profiles of different Api m 10 isoforms and variants in Api m 10-specific peptide microarrays. The data of the Api m 10 peptide arrays were normalized by calculating the Z-score and the values for the individual peptides were plotted according to the sequences of the Api m 10 isoforms. The IgE-recognition profile for serum B3 (**A**), serum E1 (**B**), and serum B5 (**C**) is presented.

(A)

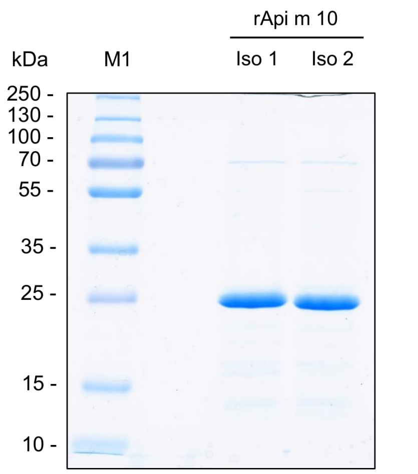

rApi m 10 isoform 1 and 2
13 % SDS-Page, reducing
0.5 µg/µl; 10 µl each
Marker 1: PageRuler Prestained Plus

(B)

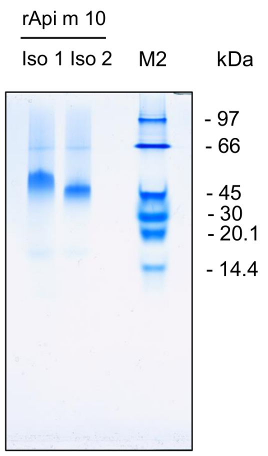

rApi m 10 isoform 1 and 2
12% native PAGE, non-reducing
0.5 µg/µl; 10 µl each
Marker 2: Low Molecular Weight Marker

Supplemental Figure 3: SDS-PAGE and native PAGE analysis of rApi m 10 isoforms 1 and 2. (**A**) rApi m 10 isoforms 1 and 2 were separated by SDS-PAGE (13%) under reducing conditions. (**B**) Dimeric states of rApi m 10 isoforms 1 and 2 were visualized after non-reducing PAGE (12%). Gels were stained with Coomassie blue.

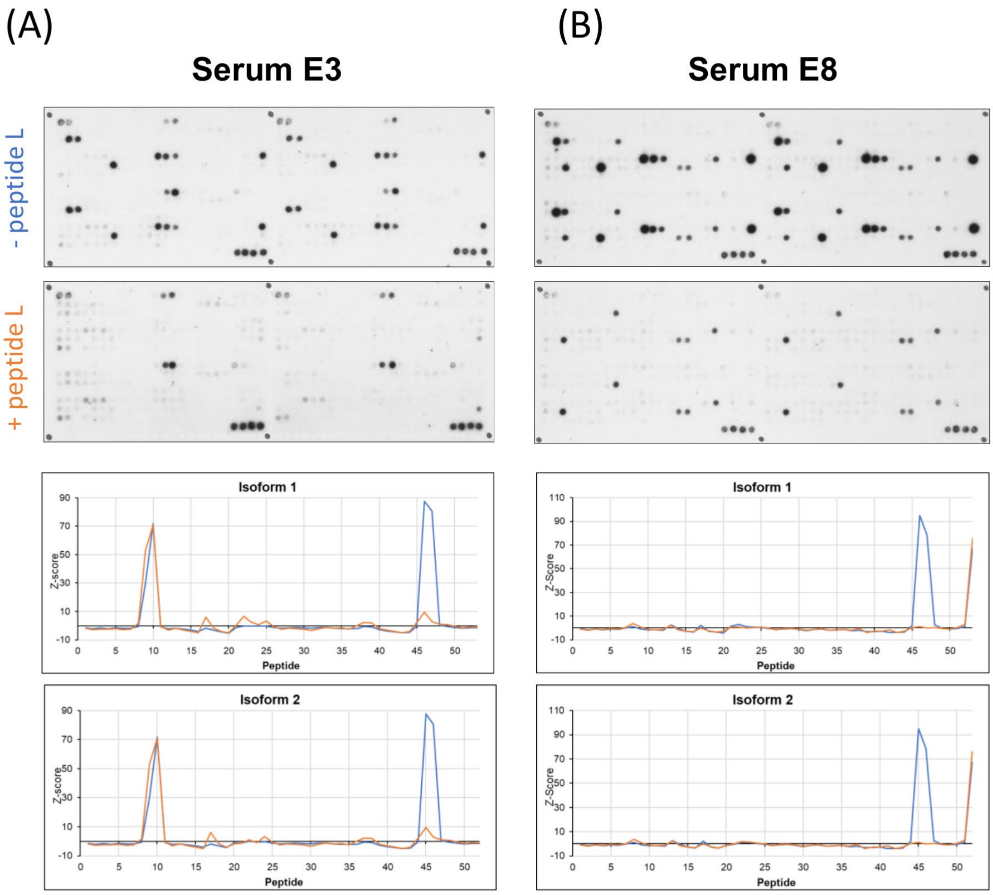

Supplemental Figure 4: Inhibition of Api m 10-peptides with IgE-reactive peptide L. Immunodetection of Api m 10-peptide arrays (above) and Z-score normalized data (below); blue curve: uninhibited; orange curve: inhibited with excess of peptide L. (**A**) Serum E3, exposure time 1 min; (**B**) Serum E8, exposure time 30 sec.

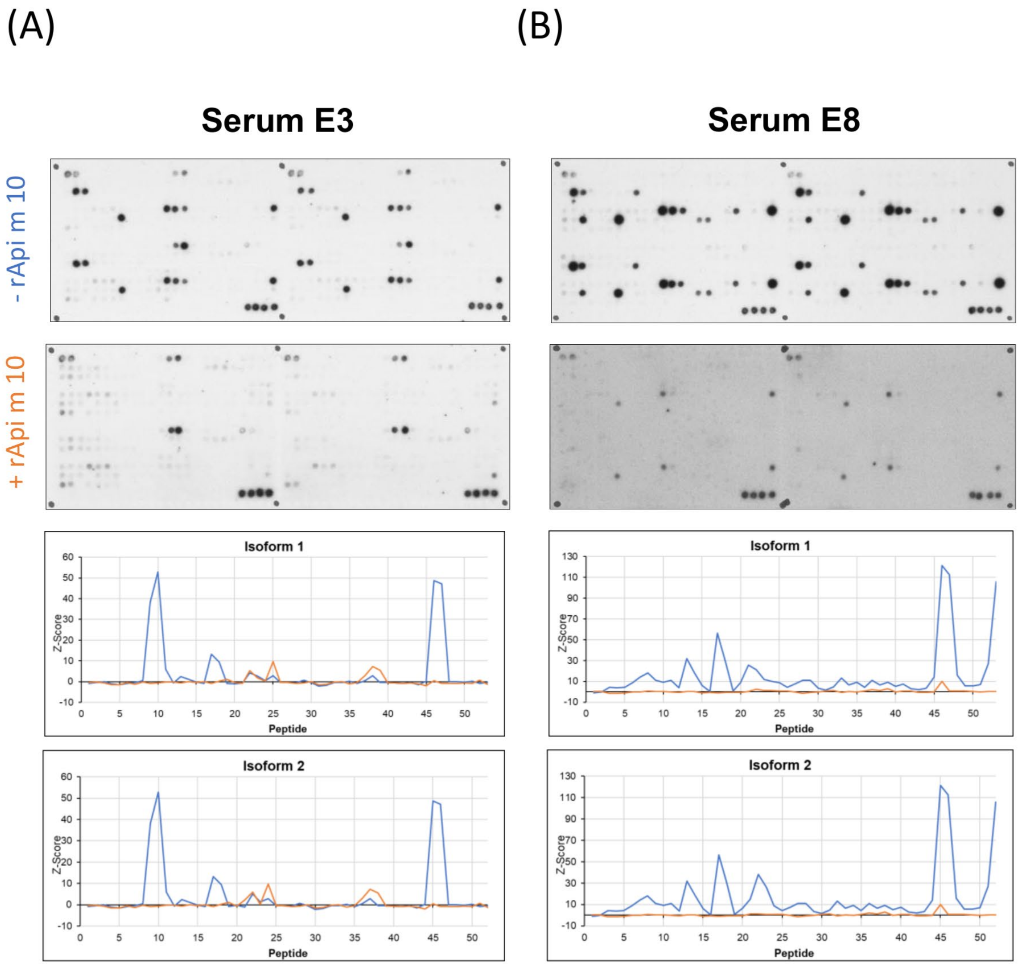

Supplemental Figure 5: Inhibition of Api m 10-peptide arrays with rApi m 10 isoform 1. Immunodetection of Api m 10-peptide arrays (above) and Z-score normalized data (below); blue curve: uninhibited; orange curve: inhibited with excess of rApi m 10 isoform 1. (**A**) Serum E3, exposure time 1 min; (**B**) Serum E8, exposure time 30 sec.

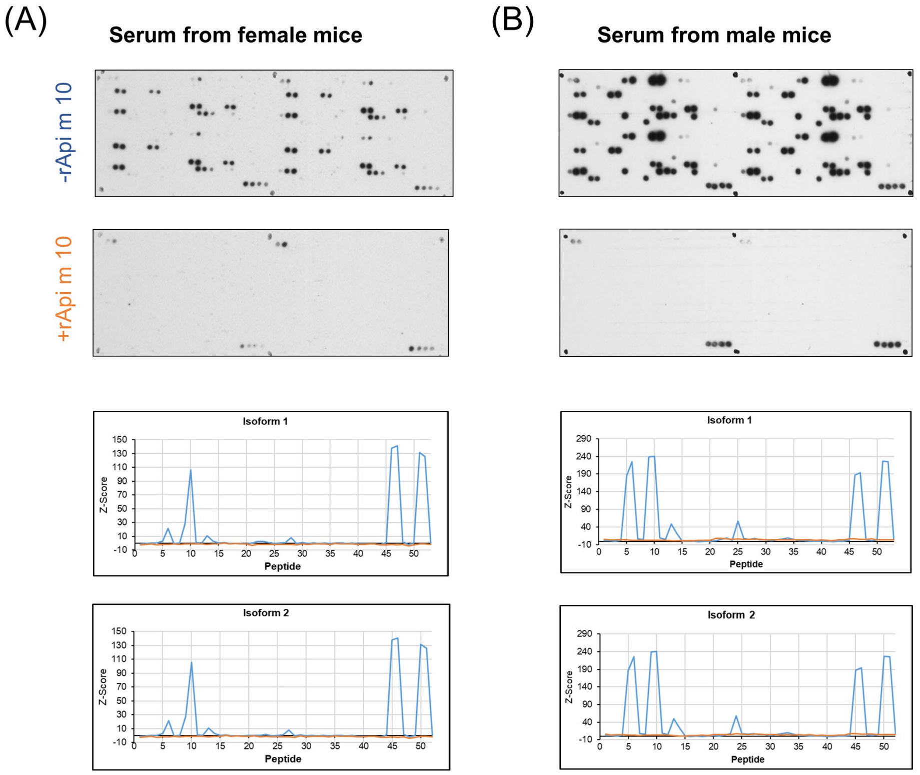

Supplemental Figure 6: Murine IgG-recognition profile in Api m 10-specific peptide microarrays (15-mers, 4 aa offset). Epitopes recognized by pool sera from (**A**) female and (**B**) male mice immunized with rApi m 10. Immunodetection of uninhibited Api m 10 peptide arrays (upper Api m 10-specific CelluSpot^{TM} slides) compared with rApi m 10 inhibition (lower slides). The data of the Api m 10 peptide arrays were normalized by calculating the Z-score and the values for the individual peptides were plotted according to the sequences of the Api m 10 isoforms from the respective N- to C-termini. The IgG-recognition profile for Api m 10 isoform 1 and 2 is presented (blue curve: uninhibited; orange curve: inhibited with excess of rApi m 10 isoform 1). (A) Exposure time 40 sec; (B) exposure time 10 sec.

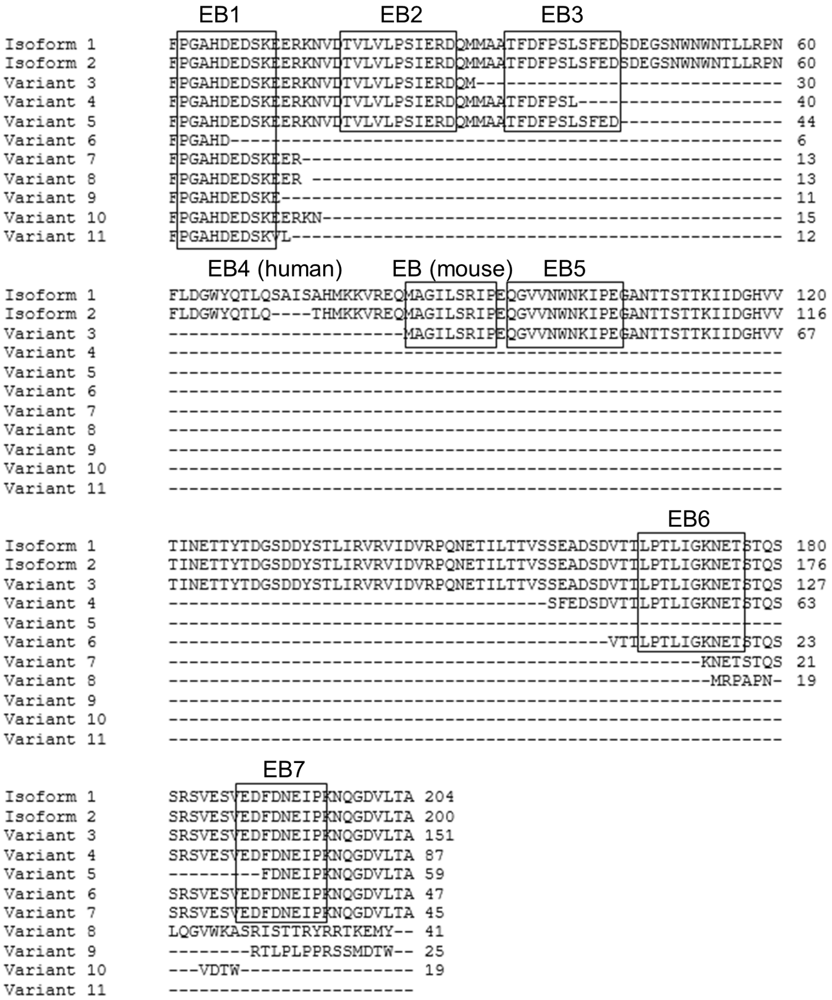

Supplemental Figure 7: IgG-reactive areas of Api m 10 variants recognized by murine pool sera. Except for EB4 (exclusively detected by human Api m 10-rective sera) serum IgG from Api m 10 sensitized BALB/c mice binds to the same epitopes that were identified as IgE-binding areas of human Api m 10-reactive sera.
